# Role of Thylakoid Lipids in Protochlorophyllide Oxidoreductase Activation: Allosteric Mechanism Elucidated by a Computational Study

**DOI:** 10.3390/ijms24010307

**Published:** 2022-12-24

**Authors:** Ruiyuan Liu, Leng Wang, Yue Meng, Fang Li, Haiyu Nie, Huizhe Lu

**Affiliations:** College of Science, China Agricultural University, Beijing 100193, China

**Keywords:** light-dependent protochlorophyllide oxidoreductase, thylakoid lipids, allosteric modulation, molecular dynamics simulation

## Abstract

Light-dependent protochlorophyllide oxidoreductase (LPOR) is a chlorophyll synthetase that catalyzes the reduction of protochlorophyllide (Pchlide) to chlorophyllide (Chlide) with indispensable roles in regulating photosynthesis processes. A recent study confirmed that thylakoid lipids (TL) were able to allosterically enhance modulator-induced LPOR activation. However, the allosteric modulation mechanism of LPOR by these compounds remains unclear. Herein, we integrated multiple computational approaches to explore the potential cavities in the *Arabidopsis thaliana* LPOR and an allosteric site around the helix-G region where high affinity for phosphatidyl glycerol (PG) was identified. Adopting accelerated molecular dynamics simulation for different LPOR states, we rigorously analyzed binary LPOR/PG and ternary LPOR/NADPH/PG complexes in terms of their dynamics, energetics, and attainable allosteric regulation. Our findings clarify the experimental observation of increased NADPH binding affinity for LPOR with PGs. Moreover, the simulations indicated that allosteric regulators targeting LPOR favor a mechanism involving lid opening upon binding to an allosteric hinge pocket mechanism. This understanding paves the way for designing novel LPOR activators and expanding the applications of LPOR.

## 1. Introduction

All the energy available for life on Earth is converted from solar energy. Chlorophyll is the main light-absorbing pigment in photosynthesis and its biosynthesis process is accompanied by high light utilization [1]. The synthesis of chlorophyll requires precise regulation of light-harvesting proteins biosynthesis [2,3,4] and associated nuclear gene expression [5,6]. Efficient coordination among chloroplast pigment synthesis, photosynthetic membrane formation [4,7], and the regulation of photoenzyme activity [8,9] mediate the progression of the photosynthetic pathway. Light-dependent protochlorophyllide oxidoreductase (LPOR, E.C. 1.3.1.33) is a key enzyme in photosynthesis, which exists in various photosynthetic organisms, such as cyanobacteria, algae, and multicellular plants [10]. LPOR gene expression directly affects the greening ability of etiolated seedlings and is essential to the growth and development of green plants [11].

**Figure 1:**
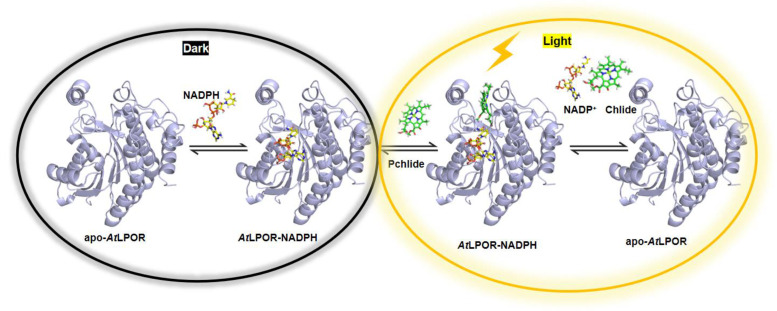
Kinetic mechanism of *At*LPOR. Note ordered binding of Pchlide substrate and NADPH cofactor.

**Figure 2:**
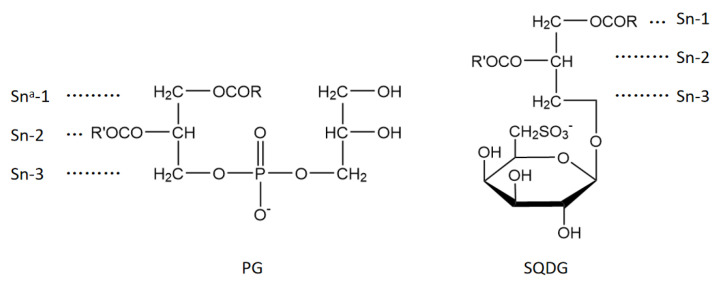
Structural formulas of phosphatidylglycerol (PG) and sulfoquinovosyldiacyglycerol (SQDG). The R groups and R’ represent various fatty acid chains of different lengths. ^a^ Stereospecifically numbering (Sn).

LPOR forms the ternary complex (LPOR/NADPH/Pchlide) in the dark followed by photocatalytic reduction under photoactivation [12] (Figure 1). In the dark, plant LPOR accumulates in immature chloroplasts or luteum, in a cubic lattice called prolamellar bodies (PLBs) that mainly consists of monogalactosyl diacylglycerol (MGDG), digalactosyl diacylglycerol (DGDG), phosphatidyl glycerol (PG), and sulfosyl quinoline diacylglycerol (SQDG) [13]. Under light, however, LPOR catalyzes the C=C reduction of Pchlide on a millisecond time scale while PLBs decompose [14]. This coupling of reductase activity and membrane dismantling is the first step in the formation of thylakoid membranes [15,16]. In plants, neutral galactolipids (MGDG and DGDG) and negatively charged lipids (PG and SQDG) constitute ca. 80% and ca. 20% of thylakoid membrane lipids, respectively [17,18]. The charged lipids PG and SQDG (Figure 2) can substitute each other under nutrient starvation conditions to maintain chloroplast functional organization. As such, their role in photosynthesis is critical [19,20,21]. At physiological pH, the negatively charged SQDG and PG may be electrostatically attracted to the LPOR complex, thus stabilizing the PLB structure [22]. Incubating isolated PLBs in low pH or ionic-containing buffers alters the photoactive LPOR complexes [23]. In addition, a significant reduction in the amounts of PGs affects the structural organization of PLBs and may change the activity of the LPOR/NADPH/Pchlide ternary complex (e.g., as in the etiolated leaves treated with amitrole and norflurazon [24]). However, although the above observations indicate a link between the activity of LPOR and the lipids in PLBs, the molecular mechanism remains unclear.

Furthermore, lipid molecules also play a regulatory role in the photosynthetic system. Through the study of photosystem II (PSII) light-harvesting pigment protein complex (LHCII), it was demonstrated that PG binds relatively strongly to LHCII and the specific binding between PG and PSII was conducive to maintaining a reasonable three-dimensional structure of the protein [25]. In addition, PGs could alter the secondary structure of proteins in PSII, leading to an increase in the number of α-helices and β-sheets along with a decrease in random coil structures, possibly due to the effect of PGs on the conformation of tyrosine residues or the surrounding microenvironment [26]. Coincidentally, experimental results (Table S1) with a similar effect of PG on LPOR were observed in a recent in vitro study. It was found that PG and SQDG enhanced the binding affinity of NADPH to plant LPOR 100-fold, which further affected the spectroscopic properties of substrates and products [27]. Collectively, these experimental studies suggest that there is specific PG-protein binding in photosynthesis, further underscoring the key role of PG in this process. Hence, to uncover the general design principles in photosynthetic enzymes, it is essential to investigate the allosteric regulation of LPOR.

In the absence of a crystal structure of LPOR in complex with a lipid molecule, we first examined the binding sites on the surface of LPOR using molecular modeling and simulation. To achieve adequate sampling, we performed several parallel conventional MD simulations of PG/LPOR, starting each simulation at a different primary binding pocket. In agreement with a recent experimental study using cryo-electron microscopy, the results suggested a relatively stable binding site [28]. An accelerated molecular dynamics simulation approach was then employed to efficiently discover long range conformational changes in LPOR. Different PGs were selected to explore their feasible binding modes with LPOR throughout the activation process. The simulation results clarified that PG binding changed the residue network distribution inside LPOR through various non-bonded interactions, resulting in an open lid by the rotation microswitches. Next, two primary binding states of PG at the allosteric site were captured by cluster analysis. Finally, the dynamic behavior of both PG and NADPH and their indirect effects on each other were analyzed by simulating the ternary complex. The results obtained in this study demonstrate the utility of computational methodologies for studying protein-lipids systems and reveal the biochemical basis for the specific interactions between LPOR and thylakoid lipids. 

## 2. Methods

### 2.1. Structural Preparation

**Figure 3:**
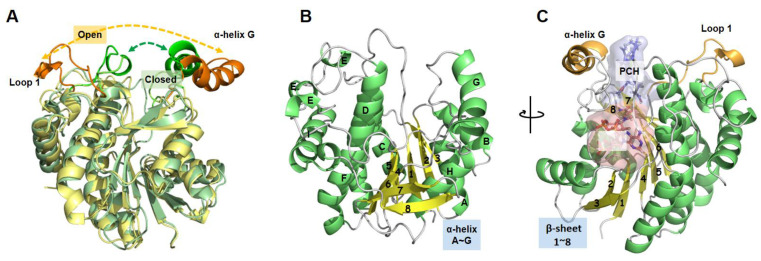
(**A**) Conformational superposition of *Arabidopsis thaliana* LPOR (PDB ID: 7JK9) and the constructed *At*LPOR in the “closed” (light green) and “open” (light yellow) state. The lid regions where the two proteins differ most are highlighted in green and orange, respectively. (**B**) The conformation of constructed *At*LPOR α-helices and β-sheets are shown in green and yellow, and connecting loop regions are shown in silver. (**C**) The constructed *At*LPOR/NADPH/Pchlide ternary complex.

There is one crystal structure from the plant *Arabidopsis thaliana* LPOR (PDB ID: 7JK9) in a “closed” state (Figure 3) in the Protein Date Bank (PDB) (http://www.rcsb.org/, accessed on 5 January 2022). Therefore, homology modeling was performed to construct an “open” conformation for better characterizing the state of the “lid” and also helping to capture the stable conformation of modulators and LPOR. 

The LPOR sequences of *Arabidopsis thaliana* were retrieved from the NCBI Blast server [29], and a structure domain search was performed for LPOR in UniProt (https://www.uniprot.org/, accessed on 5 January 2022). Since the dimeric LPOR is composed of two identical subunits, the cofactor and substrate are independent of their respective subunits and do not interact with each other. Therefore, the simulation studies were performed on one subunit. LPOR from *Thermosynechococcus elongatus* in an open conformation (PDB code: 6L1H, resolution: 2.41 Å, chain A) was selected by applying the Swissmodel server [30]. The alignment results indicated a 55.73% sequence identity between the target and template proteins, implying that the structural homology between the two proteins would be greater than 90%, ensuring the reliability of the predicted structure. To further validate the resulting model stereochemically, we employed four methods: Ramachandran diagram, ERRAT, VERIFY3D, and PROVE [31,32,33,34] (Figures S1–S4); all four methods confirmed the high stereochemical quality of the model. 

We also corroborated that the homologous proteins shared the positions of the proposed catalytic residues, as well as the NADPH location (RMSD of 3.1 Å for the heavy backbone atoms). Thus, the coordinates of NADPH were transferred directly from *T. elongatus* LPOR to the *Arabidopsis thaliana* LPOR (*At*LPOR).

### 2.2. Exploration of Potential Allosteric Sites

The specific location of the allosteric site in LPOR has not been reported yet. Since thylakoid lipids are usually present as mixtures whose hydrophilic heads tend to interact with proteins, the smallest fatty acid-free PG molecules were chosen as microprobes to explore the potential binding sites on protein surface by the following three tools.

First, the LPOR surface was analyzed to predict putative ligand binding sites using the LIGSITEcs program on a Protein Allosteric and Regulatory Sites (PARS) server [35]. Protein dynamics and structural conservation were analyzed primarily to identify pockets that may regulate ligand-binding, and ultimately obtain a ranking of their potential as allosteric sites. The *p* values were generated to evaluate whether the overall change in protein flexibility was statistically significant when the site was occupied [36]. Another server for identifying potential allosteric sites, CorrSite 2.0 [37,38], was utilized for further validaton. Unlike PARS, CorrSite 2.0 is based on the assumption that the movement of allosteric and orthosteric ligand-binding sites in proteins is highly correlated [39]. Hence, it requires manual identification of the orthosteric site and then utilizes the Gaussian network model to calculate the current presumed correlations between detected cavities with orthosteric sites, and normalizes the correlation by Z-score [40,41]. Last, the MOLCAD module in Sybyl [42,43] was also employed to locate and contour channels and cavities in LPOR. The fast Connolly method [44] was chosen to generate multiple channel surfaces because it uses a marching cube algorithm and requires less calculation time than the Connolly surface [45]. To improve accuracy, the minimum dots indicating the minimum number of surface points in a channel or cavity was set to 500. The location and the binding stability of the ligands in the hit cavities were considered as described below.

### 2.3. Molecular Docking

Most of the fatty acids in plants carry an even number of carbon atoms and can be divided into short-chain fatty acids (SCFA) with C1 to C4, medium-chain fatty acids (MCFA) with C6 to C12 and long-chain fatty acids (LCFA) with C12 and above [46]. The negatively charged PGs and SQDGs with different fatty acid chains were docked into site II in the open LPOR model and the closed LPOR structure, respectively, by the Sybyl Surflex-Dock program [47], which adopts a unique empirical scoring function to generate the binding mode between ligands and macromolecular proteins. We also performed semi-flexible docking [48] (i.e., the overall configuration of LPOR is rigid but the small molecules are conformationally variable) to accommodate the very flexible acyl fatty acid chains in lipid molecules. Before docking, the model protein was pretreated to add polar hydrogen atoms and charges. Next, the corresponding Protomols [49] were identified by the surrounding amino acid residues and then the lipid molecules were docked into the detected sites. The parameters that determined the size of cavities and the extent to which the receptor was embedded, namely, threshold and bloat, was set to 0 and 0.5, respectively. All other parameters were set to their default values.

The final docking result could generate 100 docking conformations for each ligand, and the one with highest total score was selected as the initial structure for the molecular dynamics simulation. The total Surflex-Dock score is expressed as -lg (K_d_), where K_d_ represents the ligand dissociation constant. When the total score is greater than 4, the receptor and ligand are considered effectively bound.

### 2.4. Molecular Dynamics Simulation

#### 2.4.1. Conventional Molecular Dynamics (cMD) Simulation

The deprotonated different forms of PG (0:0~18:0, 18:1, and 18:2) were modeled by the tleap [50] script of the AMBER18 software and their charges were taken from the Lipid 11 force field [51]. To obtain results that overcome the potential limitations of the force field and to further probe the allosteric mechanical, we also employed the Lipid 21 force field [52] in simulations while maintaining other settings the same. Each lipid was divided into three residues, two tail residues and one head residue, and listed sequentially following the order Sn-1 tail, head, and Sn-2 tail group. Before the simulation, the protonation states of all titratable residues of LPOR were assigned by H++ [53]. The assignments are as follows: all Glu and Asp residues were defined as negatively charged, whereas Lys and Arg were determined to be positively charged. The His319 closer to the substrate was set as HIE, which was protonated at Nε. All other histidine residues were treated as HID (protonated at the Nδ position), and all cysteine residues were considered as neutral. The amber ff14SB force field [54] was applied to treat all amino acid residues. The structures of the SQDG were preprocessed using the Antechamber module in AmberTools [55] and the DFT/B3LYP/6-31G* method in the Gaussian 16 program [56] was performed to generate the RESP charges [57]. This DFT method was further adopted to quantitatively analyze the distribution of the PG surface electrostatic potential. The complex was then dissolved into a TIP3P octahedral water model [58], 10 Å away from its boundary, providing sufficient solvent space for conformational adjustment of the docked complex. In addition, the counter ion Cl^-^ was added to keep the electroneutrality of the simulated system.

Energy minimization was carried out in three steps: first, via 2 × 10^3^ steepest descent cycles and conjugate gradient cycles [59] with harmonic force restraints of 200 kcal/ (mol·Å^2^) on solute atoms, then with an increased 5 × 10^3^ maximum number of cycles for the same algorithms as above on all atoms except for the carbon atoms in the protein backbone, finally releasing all constraints with free optimization. The system was then heated up to 310 K for a total of six times; the first four times were 2.5 × 10^3^ steps, 4 × 10^3^ steps, and then 6 × 10^3^ steps for the last two, respectively, and then equilibrated for 10^4^ steps at 310 K with a 2fs time integration step in the periodic boundary conditions. Finally, a 10 ns productive MD run was carried out in an NVT ensemble. The long-range electrostatic interactions were modeled with a particle mesh Ewald (PME) algorithm [60]. The SHAKE algorithm [61], 8 Å cutoff for nonbonded interactions, and the particle mesh Ewald method were adopted. The whole system was kept at a constant 310 K, using the weak-coupling algorithm [62] with a 0.5 ps time constant for heat bath coupling for the system. The energy information and coordinates were written every 500 steps. The coordinates written to the restart and trajectory files were wrapped into a primary box to obtain an observable structure without affecting the energy or forces.

#### 2.4.2. Accelerated Molecular Dynamics (aMD) Simulation

On the basis of the cMD simulation, the acceleration parameters were calculated for the subsequent dual-boost aMD simulation [63], and the final structure of the cMD simulation was selected as the starting structure for the aMD simulation. Systemic potential energy was calculated as $V_{r}$. The corresponding threshold $E_{thresh}$ was determined by system size and its normal energy value. During the aMD, when the instantaneous potential energy was lower than $E_{thresh}$, it would be increased for $\Delta V_{r}$ to reach $V^{*}{}_{r}$. Otherwise, the original value $V_{r}$ would be adopted (Equations (1) and (2)); $\Delta V_{r}$ depends on $E_{thresh}$ and the acceleration parameter α, and is defined according to Equation (3).

(1) $$V^{*}{}_{r}=V_{r};\;V_{r}\geq E_{thresh}$$

(2) $$V^{*}{}_{r}=V_{r}+\Delta V_{r},\;V_{r}<E_{thresh}$$

(3) $$\Delta V_{r}={\left(E_{thresh}-V_{r}\right)}^{2}/(E_{thresh}-V_{r}+\alpha )$$

A non-negative boost potential was added to both the total energy and dihedral angles across all atoms in all systems. The total boost and dihedral acceleration parameters were estimated according to Equations (4) and (5):

(4) $$E_{dihed}=V_{dihed\_avg}+\lambda \times V_{dihed\_avg}\;,\;\alpha_{dihed}=\lambda \times V_{dihed\_avg}/5$$

(5) $$E_{total}=V_{total\_avg}+0.2\times N_{atoms}\;,\;\alpha_{total}=0.2\times N_{atoms}$$

where $N_{atoms}$ denotes the total number of atoms, and $V_{dihed\_avg}$ and $V_{total\_avg}$ represent the average dihedral and total potential energies, respectively, which were computed from the 10 ns cMD simulations. λ is an adjustable acceleration parameter and was set to be 0.3. Finally, the 50 ns aMD simulation was perf ormed for each system. All MD simulations were performed using the GPU implementation of the Amber molecular simulation package [64]. 

#### 2.4.3. Unbiased Molecular Dynamics Simulation

To increase the diversity in sampling, three parallel unbiased simulations were performed for each activator at each chosen site [65,66], each of which was 25,000 ps long in an explicit solvent. The starting configurations in these three simulations differed from each other in the distribution of the initial random velocity seeds to exclude the occasional error on all atoms in the system. The snapshots were saved every 10 ps for analysis. 

The simulations were examined on a nanosecond time scale and thus may not necessarily be consistent but complementary to results reflecting protein dynamics on a microsecond to millisecond scale [67,68,69,70]. 

### 2.5. MM-GBSA Binding Free Energy (BFE) Calculation

Molecular mechanics generalized Born surface area (MM-GBSA) [71] was utilized to perform energetic postprocessing and per-residue energy decomposition of trajectories for all apoLPOR and PG/LPOR complexes in a continuous solvent model using Born radii default parameters as implemented in the igb = 2 model [72] in Amber. In the computational analysis of free energy, we chose the frames in the MD simulation before the first fundamental change for the orientation of the PG in relation to the allosteric pocket (this applies to scenarios where a PG might change its bound pose or even dissociate), which was reflected in the distance between the P atom of PG and Site II. 

For calculation, we took the frames in which the critical distance of the lipid was lower than 15 Å and was bound to the protein surface. Otherwise, all frames from MD simulations were analyzed. The trajectory selected for each system was used to estimate $\Delta G_{Total}$ according to Equation (6):

(6) $$\Delta G_{Total}=\Delta E_{\mathrm{Vdw}}+\Delta E_{Ele}+\Delta G_{Pol}+\Delta G_{Non-Pol}$$

where $\Delta E_{Vdw}$ and $\Delta E_{Ele}$ represent the van der Waals interaction and electrostatic contribution in the gas phase, respectively. $\Delta G_{Pol}$ and $\Delta G_{Non-Pol}$ are the electrostatic and nonpolar contributions of the free energy of solvation, respectively. 

### 2.6. MD Trajectory Analysis

The cpptraj module in AMBER was primarily applied to perform trajectory analysis on the MD outputs and the results were visualized by VMD [73]. The stability of the MD trajectory was assessed by calculating the root mean square deviation (RMSD). Then, based on the average position of the residues, the root mean square fluctuation (RMSF) was calculated to monitor the local conformational flexibility. RMSF calculations were performed for all frames of the MD simulation and all atoms in the analyzed molecules. The output “byres” values were calculated as the average (mass-weighted) fluctuation of each residue in LPOR. 

Distance distribution in the active site Lys332-Glu343 and Lys243-Glu379 residues of LPOR were calculated for amino N and carboxyl O atoms. Next, two dihedral angles 1 and 2 (I229/T230/G231 and 310I/311A/312T) were defined to measure the open/closed configuration of the lid (Figure S16). In this analysis, all MD simulation frames were taken into account. 

Cluster analysis using the DBSCAN clustering algorithm [74,75] was performed to reveal the representative binding modes of LPOR activators by distinguishing the structure categories from the entire simulation trajectory. 

To characterize the electrostatic properties of site II in LPOR, the electrostatic potential isosurface was calculated with a grid spacing of 1.8 Å by the Adaptive Poisson-Boltzmann Solver (APBS) program from AmberTools and the results were then visualized with the use of Pymol software [76].

The community structures of residue networks in LPOR were analyzed based on the cluster conformation by the PSN-ENM approach using webPSN v2.0 [77]. PSN-ENM employs a mixed protein structure network (PSN) and elastic network model-normal mode analysis (ENM-NMA) strategy to study the structural dynamics communication in proteins. Residue (node) communities feature sets of highly interconnected residues-residues belonging to the same community, which are densely linked to each other but sparsely connected to nodes outside the community. 

## 3. Results

### 3.1. Exploring the Potential Allosteric Sites in LPOR

Based on the predicted model, a variety of site search tools were utilized to discover potential allosteric sites on the surface of the LPOR. The predicted sites and their ranking in PARS were displayed in Figure S5A and listed in Table S2. Eight sites were detected. The first site exhibited 75.6% structural conservation and was thus highly likely to occur in structures of related protein families. However, this site was the typical Rossman fold domain (RFD) in the SDR family [78], which is the orthosteric site for cofactor NADPH binding and was therefore excluded. The conservation for the remaining detected sites was lower, possibly due to the sequence specificity of LPOR. These sites were considered statistically more likely to have potential allosteric activity the smaller the *p* value was. Orthosteric sites were excluded by Corrsite, and the cavities identified and Z scores are shown in Table S3 and Figure S5B; the top three cavities among them were considered. Furthermore, the multiple multi-channel surfaces detected method in Sybyl recognized five accessible surfaces (Table S4 and Figure S5C). 

**Figure 4:**
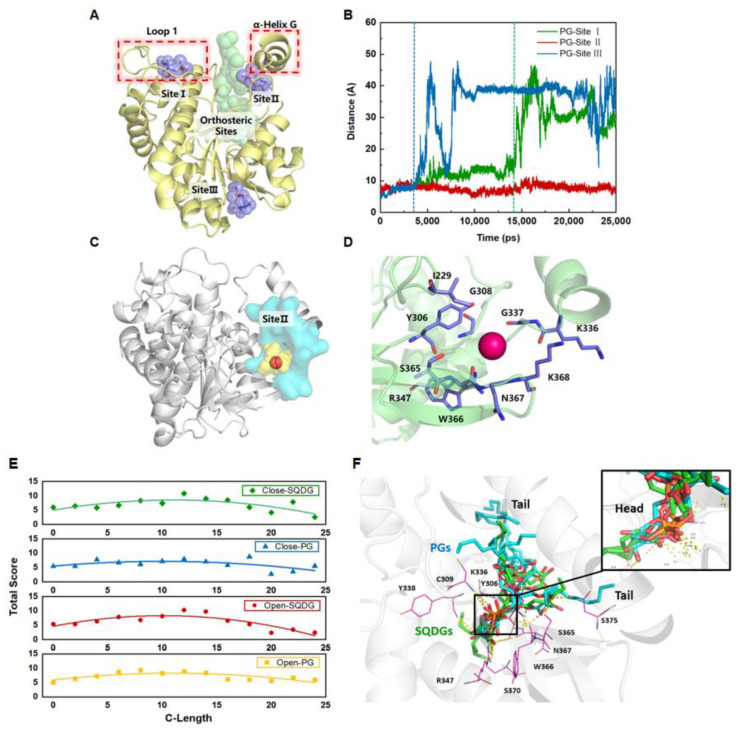
(**A**) The ternary complexes obtained by molecular docking with PGs in three potential allosteric pockets. (**B**) The distance between PG and sites I, II, and III in three unbiased molecular dynamics simulations. (**C**) The most-hit site II structure superposition. The purple ball represents the site identified by PARS, the cyan surface represents the site identified by Corrsite, and the yellow spheres represent the site identified by MOLCAD. (**D**) Amplification of site II with the key residues around. For clarity, the solvent and hydrogen atoms are not shown. (**E**) The docking scores for PGs and SQDGs to the open and closed LPOR conformations. The curves defined by dispersed coordinate points for the total score are approximated by second-order polynomial fitting. (**F**) The docking mode for PGs and SQDGs in LPOR in the open state. PGs and SQDGs are shown as cyan and green stick models, respectively, and LPOR is shown with silver cartoons. The dashed yellow lines represent hydrogen bond interactions between lipids and the neighboring residues (shown as magenta lines). The enlarged image in the upper right corner highlights the hydrophilic interaction between multiple lipid heads with LPOR protein.

In addition to the orthosteric site detected via PARS, the hit sites located too close to the substrate binding site or positioned in the extraordinarily flexible loop region were not considered. Among the sites identified, three pockets were detected by all three methods and were therefore further considered. These three pockets are depicted in Figure 4A. Since lipid–protein interplay is intently associated with the hydrophilic part of the lipid, the fatty acid chains of the PG and SQDG molecules were removed, leaving solely their hydrophilic heads as probes to dock into the three hit pockets. Three unbiased molecular dynamics simulations on the LPOR–lipids molecular systems without the fatty acid chain were then carried out separately.

From the simulation results (Figure 4B), the PG and SQDG molecules at site III (PG/SQDG_III) were observed to diffuse into the solution very quickly. Dissociation of the lipid headgroups also occurred at site I at ~14,000 ps (Table 1 and Figure S6) indicating the incompatibility between lipids and these sites. In contrast, the PG/SQDG_II system remained stable throughout the simulation. Consistent with these results, a recent study found that in AtLPOR co-crystal structures with NADPH, there was an additional weak density in the loop region above the substrate binding site, which was mainly surrounded by hydrophobic residues and ketone groups of Pchlide. It is thus likely that this density corresponds to the galactose headgroup [28]. Collectively, these results strongly support the possibility that site II is the allosteric site. Site II and its neighboring residues are shown in Figure 4C,D.

### 3.2. Molecular Docking of PGs and SQDGs to LPOR in Closed and Open States

The fitting curves of the docking scores shown in Figure 4E for four systems exhibited a certain regularity. In addition, thylakoid lipids in plants primarily incorporate PG-18C with multiple double bonds, which may affect the affinity between LPOR and ligands. However, no apparent statistical correlation was observed in the docking results for PGs with a different index of unsaturated fatty acid (IUFA) (Figure S8). The corresponding molecular docking scores for the conformations we selected ranged from 3 to 10. In Surflex-Dock used in this study, the total score reflected the magnitude of the −lg (K_d_), indicating the lengths and degrees of unsaturation of PG and SQDG had a significant impact on the adaptation to allosteric site II. However, due to the limitations of the docking algorithm and the neglect of the solvent effect, the docking results could not fully provide a structural explanation of why this fatty acid chain size was optimal in terms of ligand docking score. However, it was used as an initial structure of the LPOR–lipid complex for the molecular dynamic simulations performed under the explicit solvent model (see below).

**Figure 5:**
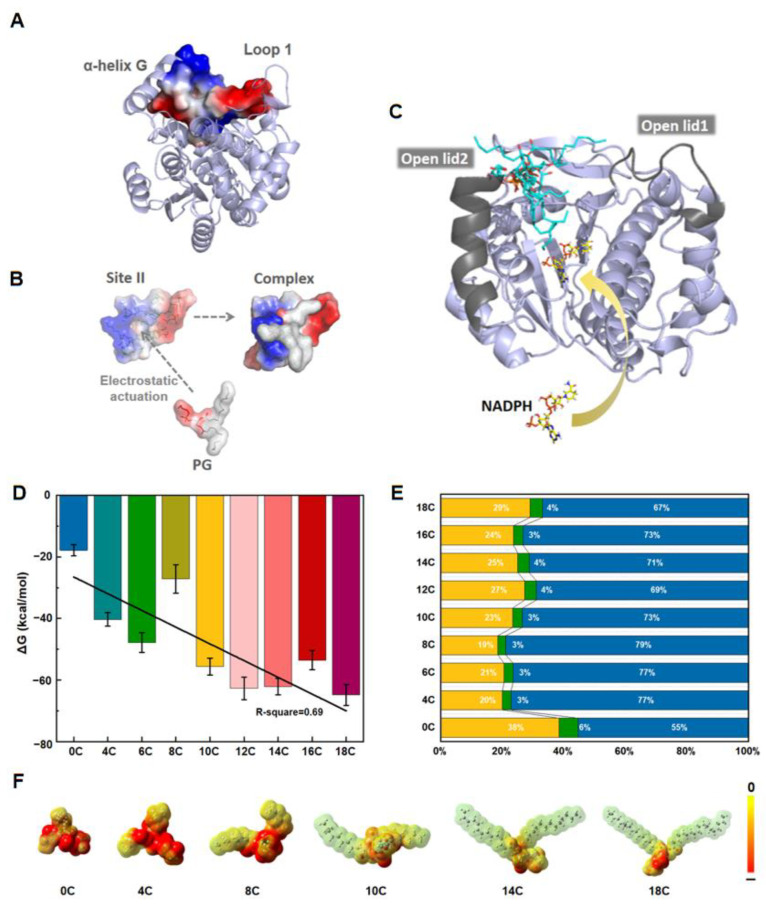
Electrostatic potential isosurface for (**A**) site II with the surrounding lid region, where red and blue represent positive and negative charges, respectively. (**B**) Schematic diagram of the electrostatic interaction driving PG to bind to site II. (**C**) PG (shown as cyan sticks) was captured to further promote the NADPH binding by affecting the lid conformation. (**D**) The total energy of LPOR binding PGs with different lengths. (**E**) Contribution of the energy term for favorable binding in nine systems (Ele: blue, Vdw: green, and Non-Pol: yellow, respectively). (**F**) The distribution of the electrostatic potential energy for different PG molecules on the electron density isosurfaces. The negatively charged nature of the PG head group caused the electrostatic potential energy of each part to be negative.

It should be noted that site II was spatially close to the “lid” region of LPOR. This “lid” consisted of two parts, lid1 (α-helix G) and lid2 (loop1) [79,80], which were flanked by the upper region of the active site and formed a major hydrophobic opening. Its conformational change played a key role in the binding of the LPOR substrate and cofactor, and was highly correlated with the photocatalytic rate of LPOR [81]. A closer inspection of the binding site showed that the binding site of the PG headgroup was rich in polar residues, giving rise to the formation of many hydrogen bonds (Figure 4F). These hydrogen bonds may be essential to establish stable binding interactions while the hydrophobic tails point in the direction of the solvent environment. The electrostatic potential surface (Figure 5A,B) revealed that the binding between the negatively charged head group of PGs and the region with positive electrostatic potential in lid1 stabilized the local charge distribution. Therefore, we speculated that the binding of lipids to site II may affect the conformation of the lid area and facilitate NADPH binding to LPOR by keeping the α-helical G (lid1) in a relatively stable state in an allosteric manner (Figure 5C). However, due to the flexibility of the non-polar tails of these lipids, the mechanism of their binding to proteins remains to be investigated.

### 3.3. PGs with LCFA Bind More Favorably at the Site II through Noncovalent Interaction

The orientation or polarity of PGs plays a key role in the specificity of lipid–protein interactions, as observed in studies combining NMR experiments and molecular models [82,83,84]. Hence, we next performed aMD simulations for different complexes of LPOR–PGs to investigate the potential dependence between the length of the PG acyl chains and the binding stability. The simulation results suggested that the LPOR–PG binding was energetically unfavorable in most cases with the PGs eventually dissociating from site II (Figure S13). The time the PGs remained at site II for each system (Table S6) was characterized based on the distance of the P atom of the PGs from the residue at the site center. A distance larger than 8 Å was used to indicate the occurrence of dissociation. Before the critical point of dissociation was reached, the total binding free energies (∆BFE) as well as the energy decomposition for the interaction between PG and LPOR were calculated for all systems.

The results of this analysis suggested an increased affinity of PG to LPOR with the lengthening of the carbon chain in PG, with a correlation coefficient R-squared value of 0.69 (Figure 5D). Furthermore, among the various driving energy terms, the electrostatic interaction energy contributed the most to the binding, exceeding 50% of the total energy in each system (Figure 5E). This is mainly due to the hydrophilic heads of the PGs as well as their negatively charged nature. With the extension of PG tail, no significant variations were discovered in the polar solvation and electrostatic interaction strengths. However, the van der Waals contributions and non-polar solvent contributions gradually increased, possibly due to the diminished polarity and the redistributed charge in the PG molecule (Figure 5F). The binding mode of the PG molecule further changed to make it more conducive to binding in the hydrophobic cavity of LPOR. In the absence of the acyl chains, the ∆BFE of the PG headgroup was less than −20 kcal/mol, indicating that the fatty acid chain of PG was indispensable for binding. In terms of the electrostatic interaction energy, the contribution of arginine and lysine to binding played a major role in each system (as shown in Figure S9). For the van der Waals interaction energy, LPOR residues concentrated around the lid area made the primary contribution to the interaction with the PG molecule (Figure S10). In the absence of the acyl chains, however, these active residues made little contribution (PG-0C) (Figures S9, S10 and S11).

These results suggest that PG binding activated the orthosteric pocket channel by interacting with the lid area to facilitate communication among active residues. These effects may further modulate the conformational changes of LPOR to efficiently capture the cofactor. 

### 3.4. The Lid Opening Is Achieved through a Hinge with Salt Bridges

LPOR belongs to the short-chain dehydrogenase/reductase (SDR) family, which has a typical RFD with six central β-strands and two pairs of α-helices, forming a β-α-β-α-β topology. It is a highly conserved site for nucleotide binding and its conformational changes correspond to LPOR activity. Therefore, the stability of the RFD in the simulation was assessed by calculating the RMSD. The aMD results of LPOR-PGs demonstrated that the binding of PGs was unstable in multiple systems, and some tended to leave site II (Table S6, Figure S13A), possibly due to the enhanced interaction between large hydrophobic side chains and the solvent. Since the activation effect of PG-10C binding was significantly higher than that of the other systems (Figure S13B), we carried out further sampling.

**Figure 6:**
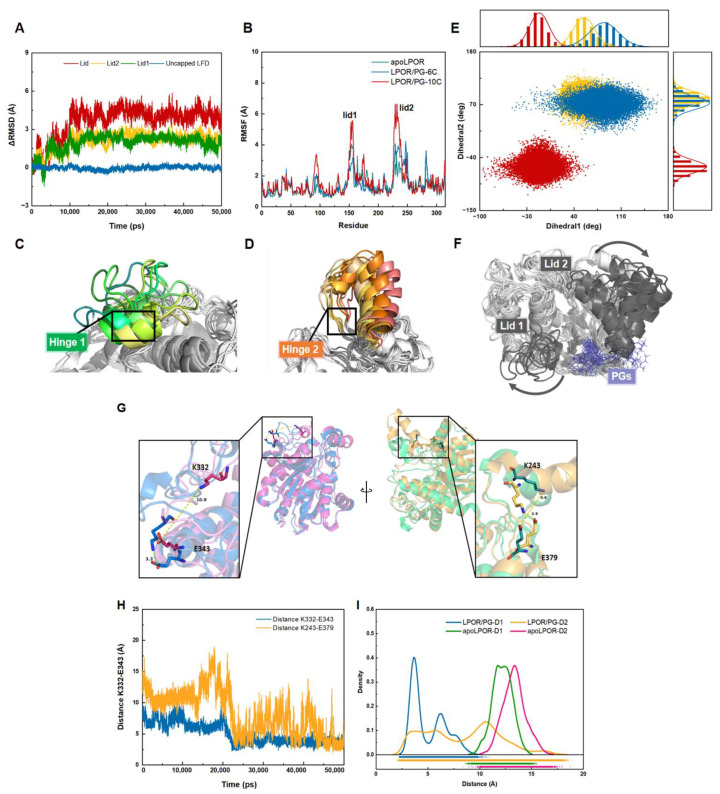
(**A**) Changes in the RMSD values of the lid relative to the RFD domain in the LPOR/PG structure during the simulations. (**B**) RMSF plots of all the atoms of LPOR/PG and apoLPOR. Structure data of lid1 (**C**) and lid2 (**D**) colored according to the opening pathway progress from yellow to green and orange to red, respectively. (**E**) Sampling of variables quantifying the lid position in aMD simulations of closed (red), open (blue) apoLPOR, and the open LPOR/PG (orange) systems. Dots represent MD snapshots in the last 20 ns runs. The variables are defined as two dihedral angles, representing the flipping of the I229-T230-G231 and 310I-311A-312T rotary microswitches in the hinge region. The details are shown in Figure S16. (**F**) The integral opening process shown to emphasize the hinge and lid motion in the simulated close LPOR structure, viewed from above. The black arrows display the movement direction of the lid in the simulations with respect to the inactive closed state. (**G**) Switching of the two salt bridges in the representative conformations of the LPOR/PG systems. The yellow dotted line represents the length of the salt bridge. (**H**) Comparison of the distances between the N atom in the side chain of K243/332 and O atom in the side chain of E379/343 for the LPOR/PG systems. (**I**) Distributions of distances 1 and 2 of Lys332-Glu343 and Lys243-Glu379 during the 50 ns simulation time for closed apoLPOR and LPOR/PG. The green, pink, blue, and yellow solid lines denote D1 and D2 in the closed crystal structure (PDB ID: 7JK9) and open structure of LPOR, respectively.

During the simulation, the “lid” structure in LPOR in the bound state was lightly flexible but tended to form an over-closed state (Figure S15) in apoLPOR. Notably, a pair of triple-residue hinges connected the lid (Figure S16) to the rest of the protein, allowing a large conformational transition while maintaining the relative rigidity of the rest of the structure (Figure 6A). PG binding not only significantly increased the flexibility of the lid region, but also adjusted the rest of the protein, resulting in a more uniform LPOR conformation. In addition, the RMSF value of the hinge residues increased significantly, indicating the activation of LPOR after PG binding (Figure 6B). This phenomenon was supported by experimental studies showing that the TT316/317AA LPOR mutant was inactive even at very high NADPH concentrations in the absence of thylakoid lipids [27]. Moreover, in the simulation of PG-10C in close-LPOR, the dominant movement we observed in loop1 of lid1 and α-helix G of lid2 was a progressive opening and expansion process (Figure 6C,D,F). However, in the absence of PG, throughout the aMD simulation, all lids in apoLPOR in the open and closed states remained in their respective conformations, with no spontaneous transitions between open and closed LPOR (Figure 6E). After capturing PG, the lid eventually transitioned to an open conformation from the initial closed state. These results demonstrated that the regulation of PG-10C binding site II near the lid region was consistent with the crucial mechanistic role of the hinge region in LPOR activation.

Furthermore, two highly conserved residue pairs (Lys332-Glu343 and Lys243-Glu379) [79] located in both sides of the lid area tended to form salt bridges during the simulation. The distances between the two pairs of amino and carboxyl groups were 9.6 Å and 10.9 Å, respectively, in free LPOR (Figure 6G), supporting the salt bridge in a dissociated state. However, as the simulation proceeded, the distance distribution of the two salt bridges gradually decreased to ~5 Å, corresponding to the transition from the closed to the open lid state (Figure 6H,I). Hence, it is likely that the two salt bridges formed only when the lid was open and the electrostatic interaction may be the intrinsic driving force behind the hinge motion, supporting a mechanistic function for this region.

This suggests that the binding of ligands in the allosteric hinge pocket can preferentially stabilize the lid in a more open conformation to modulate RFD dynamics. In particular, hinge binders can serve as a valuable rotary microswitch to characterize the opening angle of the lid. Furthermore, the formation of salt bridges revealed the mechanism for lid opening, supporting the hypothesis that the hinge region can regulate lid opening, underscoring the coupling of lid configuration and protein activation.

### 3.5. Dynamic Simulations Reveal Two Modes of Action between PGs and LPOR

**Figure 7:**
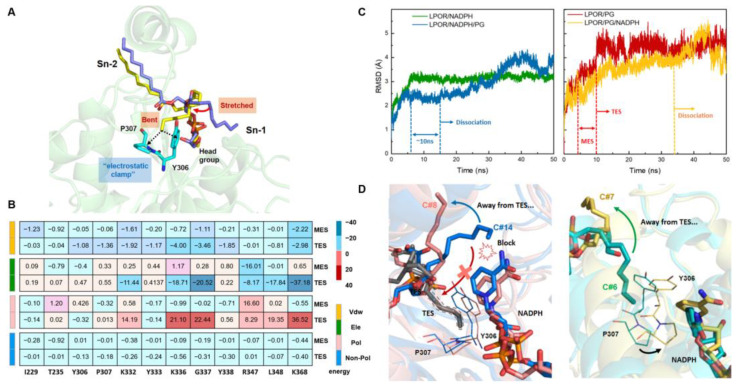
(**A**) The conformational transition extracted in LPOR/PG simulation. The PG is shown in stick with blue and yellow in MES and TES conformations, respectively. (**B**) The heat map of the decomposition of LPOR/PG in the binding pocket. The shades of red and blue represent the magnitude of BFE, respectively, and the energy items of electrostatic energy (ele), van der Waals (vdW), nonpolar, and polar contributions to solvation (sol) are shown in different color. (**C**) The RMSD curves for two binding orders. The NADPH (left) or PG (right) preferentially combined with LPOR, respectively. (**D**) Superimposition of the binding mode represented by PG binding mode cluster in LPOR/NADPH/PG (left) and in LPOR/PG/NADPH (right) with different conformations. Below each binding mode illustration, the ranking number of the binding mode corresponding to (Table S11 and S12) are listed. The TES conformation is obtained by manual alignment as a reference.

In MDs, the PG-10C stayed at site II throughout the simulations and the LPOR/PG-10C system presented the largest RMSF values compared to other systems (Figure S13C). The RMSD values of LPOR/PG-10C indicated that the complex maintained a metastable equilibrium state (MES) for 6 ns and reached the true equilibrium state (TES) after 10 ns (Figure 7C). The dominant structures in these two equilibrium states were extracted by cluster analysis (Figure 7A) and their dynamic changes were further captured. During the simulation, the hydrophilic headgroup of the PG molecule and the fatty acid chain at Sn-2 invariably stabilized the binding of the lipid to the α-helix site, forming van der Waals interactions with Lys332 and Glu343, possibly facilitating the formation of a salt bridge. On the other hand, the fatty acid chain at Sn-1 underwent significant conformational changes. The first conformational transition involved rotation and gradual stretching from bending. Then, the fatty acid chain gradually approached the lid2 region of LPOR by flipping, corresponding to the free state MES. After 10 ns, it further changed from the extended long-chain state to a curved conformation, and was stably adsorbed into the inner wall of the LPOR cavity. This complete process was captured as depicted in the Supplementary Video and was reflected by the decrease in the contribution of van der Waals energy for the corresponding residues (Figure 7B). 

Next, we analyzed two trajectory segments, before and after 10 ns. In TES and MES, the aromatic residues Tyr306 and Pro307 exhibited the greatest increase in van der Waals contributions, responsible for an “electrostatic clamp”. It formed a C-H…π interaction with the carbon end in the Sn-1 chain of PG. The fixation of the carbon chain also supported the binding of other parts of PG, reflected by the enhancement of electrostatic energy contribution of K336 and K368. Furthermore, the dominant residues bound to PG were very close to the 310I-311A-312S rotary microswitch. It was reasonable to believe that the effect of the interaction between PG and the hinge region of LPOR would propagate to another area, promoting changes in LPOR structure. PSN analysis indicated that Y306 acted as a hub with five links to other residues (Figure S18), suggesting the predominance for Y306 in intra-protein communication. Moreover, the number of communities in the PRNA of the binary complex was reduced to 12, and the hubs increased to 18 (Figures S19 and S20). This greatly increased the number of pathways within LPOR and shortened the length of the pathway necessary to facilitate protein activation. 

Therefore, after PG acted as a positive allosteric modulator to trigger the activation site on the surface of the LPOR, the effect was further transmitted to other areas through the residue network in LPOR, thereby promoting signal transduction and realizing the regulatory pathway of LPOR.

### 3.6. Cofactor Behavior and Bound PG Form a Feedback Loop by Competing for the Electrostatic Clamp

Next, we carried out simulations of the LPOR/NADPH/PG ternary complex system. The binding order was unclear since it involved two different molecules bound to LPOR. When NADPH was preferentially combined with LPOR, PG was added to simulate the ternary complex after the LPOR/NADPH binary complex reached equilibrium. The RMSD calculation results (Figure 7C) showed that the LPOR/NADPH/PG ternary complex system took longer to stabilize than the binary complex system, reaching a more unstable state. Extracted by cluster analysis, the relatively diverse composition (Table S11) of the complex conformations during this process corresponded to the dissociation of the modulators from site II (Figure S22A). This illustrated that the modulators had a negative effect on the stability of the system. In addition, due to the presence of NADPH, the acyl fatty acid chains in PG were unable to complete the conformational transition from MES to TES (Figure 7D), preventing PG from stably binding to LPOR and resulting in further dissociation. However, the steady state of ~10 ns corresponding to MES in the simulation could explain the observation that LPOR still formed a relatively stable complex in the presence of PG, indicating that PG enhanced the binding of NADPH to a certain extent.

Accordingly, PG binding resulted in a conformational change at the hinge site of LPOR, leading to the opening of the lid and facilitating the entry of NADPH. However, since only the catalytic reaction could take place in closed lid conformation [80], we next investigated whether PG binding would affect the subsequent reaction process. To this end, an LPOR/PG/NADPH system was constructed by reversing the binding order of the two molecules, and an MD simulation was performed. In this system, the dissociation phenomenon of PG was also observed, but PG stayed at the original site for a longer period of time. The LPOR/NADPH/PG system lost its original conformation quickly, whereas in the LPOR/PG/NADPH, the PG molecule slowly left the binding site and the process lasted longer (Figure S22). This result was in agreement with the previous conclusion that PG tended to dissociate from site II or did not even bind to the protein when NADPH was bound to the LPOR/PG complex. Additionally, it indicated that the TES was more stable than MES, which was further supported by the results of ∆BFE calculations (Table S10). 

As NADPH bound to the LPOR/PG complex, the measured opening length of lid was reduced from 25 Å to less than 20 Å (Figure S15). In this situation, the lid region no longer remained largely open and NADPH binding could attenuate the interaction between PG and the α-helix in the lid region. Notably, the dissociation process of PG completely fit with the putative reverse binding process from MES to TES. Following the NADPH binding, Tyr306 and Pro307 moved to the nicotinamide ring of NADPH and disrupted the C-H…π stacking interaction with PG (Figure 7D). This competitive binding of NADPH and PG to the electrostatic clamp destabilized the interaction between LPOR and PG, resulting in the dissociation of PG. At the same time, however, PG could keep the LPOR lid open in the MES, possibly facilitating the binding of NADPH. Furthermore, the RMSD values of the LPOR/PG/NADPH system fluctuated more than the LPOR/NADPH/PG, revealing that PG tended to activate the protein only when NADPH was not present in the system. 

Both cases signified that NADPH binding would deactivate the protein system, i.e., that PG only acted as an allosteric modulator and did not affect the normal catalytic process. These results suggest a feedback regulation mechanism involving PG and NADPH. Accordingly, PG enhanced the binding of NADPH, and conversely, PG would dissociate from LPOR due to cofactor interference after performing its regulatory function. 

### 3.7. Comparative Analysis of LPOR/PG Complexes in aMD Simulations with Different Force Fields

To further validate of the conclusions, we compared the simulation results using different force fields. MD simulations of the three LPOR-PG complexes (LPOR-PG_I, II and III) were performed with a Lipid21 force field. All other simulation settings were the same as those used with Lipid 11. As the results in Figure S7 and Table S5 show, after changing the force field, there was no significant change in the residence time of PG in site III, and while the PG_I system remained stable for an extended period of time, a dissociation ultimately occurred. Notably, the PG_II system exhibited the most stable binding in both simulations. 

Next, we explored the dynamic conformational changes in LPOR in LPOR/PG-10C system under the Lipid21 force field. We first examined whether the simulation reasonably converged for the LPOR/PG-10C complexes. As shown in Figure S14, the RMSD curves of the complex system could be quickly equilibrated in the TES state within the initial 10~15 ns under the two force fields. However, the RMSD in the simulation using Lipid11 underwent a larger fluctuation throughout the simulation, possibly due to the orientation adjustment of the side chains of the active residues. Analyzing the conformational change and RMSF, we found that the remarkable increase in the RMSF value of the LPOR-PG complexes was mainly due to the conformational changes in lid1 and lid2. The opening progress of the lid region was captured under both force fields by extracting the simulation conformations, albeit the effect observed under the Lipid11 force field was more pronounced (Figure S17). 

In the aMD simulations under both force fields, PG-10C was steadily packed in Site II. We further extracted the final conformations of the simulations under the two force fields to analyze the binding modes of PG-LPOR. As shown in Figure S21, the binding modes of the lipid head to LPOR differed slightly, which may be attributed to the alteration of the lipid head group torsion parameters in Lipid 21 [52]. Importantly, similar to the results of the simulations using Lipid 11, the predominant head conformation was bound to LPOR by a hydrophilic force, and the same key interacting residues were identified in the calculation of the energy decomposition (Table S9). In addition, the dominant C-H…π interaction between the electrostatic clamp (Y306-P307) and the carbon end in the Sn-1 chain of PG was observed using Lipid21, consistent with the result of the simulations using the Lipid11 force field.

To further characterize the binding affinities of LPOR with different PGs, the binding free energies (∆BFE) of the complexes were calculated with the two force fields. These calculations from independent MD trajectories using two different force fields yielded consistent results (Table S8). The electrostatic energy offset the polar solvation contributions, whereas the van der Waals and non-polar solvation energy both contributed to the binding. The results confirmed that the affinity of PG to LPOR increased as the carbon chain of PG extended. In addition, the per-residue free energy contributions in LPOR/PG-10C system were calculated and are displayed in Figure S12 and Table S9. The binding free energy of four residues (Ile229, Tyr306, Lys336, and Lys368) was found to have the highest binding contribution under both force fields. The ∆BFE calculated in the Lipid21 force field was lower, corresponding to the more stable RMSD value. Despite the unfavorable polar solvation energy of key residues, the electrostatic energy values were as high as −49.13 kcal/mol, especially for Lys368. Its side chain amino group was involved in the formation of significant hydrogen bonding interactions with the phosphate and hydroxyl group of the PG molecule in both force fields.

## 4. Conclusion and Discussion 

Understanding where and how lipid molecules bind to LPOR is essential for uncovering how these small-molecule regulators activate LPOR. To avoid the bias caused by subjective selection, our MD simulations of each LPOR regulator started with three different feasible sites. The results suggested that all regulators prefer site II, and this observation was insensitive to the selection of force fields. This result was also consistent with the weak density observed in *At*LPOR co-crystal structures with NADPH [28]. The relatively flexible helix-G and Loop1 structures around site II stabilized the binding of lipids such as PG to LPOR. The amphiphilic properties of PGs determine their affinity to LPOR; however, there were differences in the binding modes of PG molecules that differed in their acyl chain length. In addition to the headgroup of PG that formed hydrogen bonds and electrostatic interactions with LPOR, the non-polar tail could not be ignored. An increased acyl chain length corresponded to an increased contribution of the hydrophobic interactions to the binding energy.

Our MD simulations of the LPOR/PG were started from their docking poses obtained using an LPOR structure with a closed or open lid. The activated state of LPOR may be reached more directly when the MD simulations are started from an open lid, but such simulations will not reveal the dynamic details that occur during the activation process. We therefore based the molecular docking and the subsequent MD simulation on the closed LPOR structure. The nanosecond-scale aMD simulations revealed that PGs might not only play an important role in the conformational change of the lid region, but also be crucial for preserving an appropriate open conformation of LPOR with the active site accessible. The location of a hinge region that flexibly controlled lid conformational changes provided a plausible explanation for the experimental observation of LPOR inactivation by the TT316/317AA mutations in the absence of thylakoid lipids [27]. In addition, it was the formation of two pairs of ionic locks that drove the conformational transition through the hinge region. We also found that the tail of PG formed a C-H…π interaction with Tyr306 and Pro307 of LPOR, which gradually unraveled after NADPH binding. The simulation results in different force fields further supported the potential role of these key residues in the formation of the LPOR/PG complexes, and it was critical to analyze their corresponding binding contribution. In addition, a negative feedback regulation mechanism was realized. Accordingly, PG plays a role in promoting the binding of NADPH to LPOR, and the cofactor, in turn, drives the dissociation of PG due to the competition for electrostatic clamps. PG binding can reduce lid closing and stabilize intermediate states. Hence, the orientation or polarity of PGs may facilitate the investigation of the coupling between lid opening and LPOR activity, thought to be the crucial link between membrane binding with LPOR. 

In the absence of experimental validation of the conclusions, it is critical to choose an accurate force field. To this end, we compared the results of simulations obtained using the Lipid 11 and Lipid 21 force fields. The simulations using the Lipid 11 force field captured the more notable kinetic conformational changes of the LPOR/PG complex system, while the simulations using the Lipid 21 force field exhibited slightly lower energy values. The results for the primary binding modes and quantitative binding affinity were highly consistent under the two force fields. These comparative studies not only support the binding mechanism but also demonstrate an efficient approach for performing theoretical simulations of protein LPOR and PGs.

Collectively, the findings presented in this study provide a detailed and systematic description of the interactions between LPOR and PGs, uncovering the mechanism underlying the LPOR activation process. These results set the stage for developing novel plant modulators in the field of sustainable agriculture.

## Figures and Tables

**Table 1:** Behavior of the two LPOR regulators at each binding site during the MD simulation.

Binding Site			Residence Time (ps)			
	PG			SQDG		
	1	2	3	1	2	3
I	12,301	10,546	13,147	10,980	14,011	13,753
II	>25,000	>25,000	>25,000	>25,000	>25,000	>25,000
III	2230	2317	3793	1224	1329	2957

## Supplementary Materials

The following supporting information can be downloaded at: https://www.mdpi.com/article/10.3390/ijms24010307/s1, Table S1: The effect of the lipids on the Pchlide and Chlide emission maximum and the secondary structure composition of LPOR; Table S2: Results of Cavity Prediction by PARS; Table S3: Results of Cavity Prediction by Corrsite; Table S4: Results of Cavity Detection by MOLCAD; Table S5: Behavior of the PG at Each Binding Site during MD Simulation; Table S6: The Calculated Time that PGs remained stable at site II; Table S7: The Distribution of The Key Interaction Residues with PG in Site II; Table S8: Binding Free Energies for the LPOR-PG Complexes Calculated from Independent MD Trajectory with Lipid11 and Lipid21 Force Fields; Table S9: Decomposition for the Important Residues Contributing to the Binding Free Energy; Table S10: The MM/GBSA binding free energy calculation for PG with different states; Table S11: Basic information of the final clusters of the LPOR/NADPH/PG structures obtained from the aMD simulation; Table S12: Basic information of the final clusters of the LPOR/PG/NADPH structures obtained from aMD simulation. Figure S1: Ramachandran Plot of (A) template protein (PDBID:6l1h) and (B) predictor protein; Figure S2: ERRAT of (A) template protein (PDBID:6l1h) and (B) predictor protein; Figure S3: VERIFY3D of (A) template protein (PDBID:6l1h) and (B) predictor protein; Figure S4: PROVE of (A) template protein (PDBID:6l1h) and (B) predictor protein; Figure S5: Results of Cavity Prediction by (A) PARS, (B) Corrsite, and (C) MOLCAD; Figure S6: The distance between two LPOR regulators and each binding site during the MDs time; Figure S7: The distances between PG and site I, II and III in molecular dynamics simulation with Lipid 11 (A) and Lipid 21(B) force fields; Figure S8: The constructs of PGs with a different index of unsaturated fatty acid (IUFA) before docking (A); Docking results for different PGs to open and closed LPOR (B); Figure S9: Energy decomposition in nine systems. The electrostatic and polar solvation energy are shown as red and yellow line, respectively; Figure S10: Energy decomposition in nine systems. The van der Waals and Non-polar solvation energies are shown as blue and green lines respectively; Figure S11: Decomposition of total energy in nine systems; Figure S12: Total binding free energy contributions of LPOR-PG complexes with different force fields; Figure S13: The distance (A) between PG and site II in aMD; The RMSD (B) and RMSF (C) curves for LPOR/PGs systems; Figure S14: The time-evolution RMSD (A) and RMSF (B) curves of LPOR and LPOR-PG complex under Lipid11 and Lipid21 force fields; Figure S15: Superimposed views of open and closed LPOR (A); The change in the distance in 50ns simulation(B); The change in the distance in binary (LPOR/PG) and ternary (LPOR/PG/NADPH) complexes (C); Figure S16: Conformational flipping of the I229-T230-G231 (A) and 310I-311A-312T (B) rotary microswitches at the initial and final states in the simulations; Figure S17: The integral opening process shown to emphasize the hinge and lid motion in the simulated close LPOR structure under two different force fields; Figure S18: The community(left) and hub(right) structures of residue contact networks in (A) unbound LPOR and (B)LPOR bound with PG; Figure S19: The community of residue contact networks in unbound LPOR and LPOR bound with PG; Figure S20: The hub of residue contact networks in unbound LPOR and LPOR bound with PG; Figure S21: Key interactions at the active sites of the representative conformations of LPOR-PG complexes with equilibrium stabilization in Lipid11 and Lipid21 force fields; Figure S22: The extracted conformation in the two simulations. The NADPH (A) or PG (B) preferentially combined with LPOR respectively. Video S1: Dynamic conformational change from MES to TES of LPOR/PG.
