# Supplementary material for: Role of Thylakoid Lipids in Protochlorophyllide Oxidoreductase Activation: Allosteric Mechanism Elucidated by a Computational Study
Source: Int J Mol Sci. 2022 Dec 24;24(1):307. doi: 10.3390/ijms24010307 (PMC9820216; doi:10.3390/ijms24010307)
Supplement: Supplementary file 1 [file ijms-24-00307-s001.zip › Supplementary Information.pdf]

Supplementary Information (SI)

1. Supplementary Tables

Table S1. The effect of the lipids on the Pchlide and Chlide emission maximum and the secondary structure composition of LPOR.

System	Pchlide Emission maximam/nm	Chlide Emission maximam/nm	Additives	change in the Secondary structure composition/%			
				helix	sheet	turn	unord
LPOR-NADPH (L)	636	677	NADPH	-0.24	-1.32	-0.02	+1.61
LPOR-NADPH (L)-PG	647	678	PG	-2.76	+2.25	+1.24	+1.62
LPOR-NADPH (L) -SQDG	643	679	DGDG	-3.25	+1.82	+0.20	+1.14
LPOR-NADPH (H)	646	682	MGDG	-3.02	+1.85	+0.77	-0.02
LPOR-NADPH (H) -PG	647	686	TL	-3.59	+2.25	+0.11	+0.02
LPOR-NADPH (H) -SQDG	648	685	Pchlide	-2.84	+1.82	+0.54	+0.02

Table S2. Results of Cavity Prediction by PARS.

Cavity	Flexibility P-Value	Structural Conservation
1[a]	0.01	75.60
2	0.52	1.20
3	0.91	0.80
4	0.39	1.50
5	0.72	14.80
6	0.35	1.50
7	0.46	14.80
8	0.79	14.10

[a] The cavity highlighted in red is the excluded orthosteric site.

Table S3. Results of Cavity Prediction by Corrsite.

Cavity	Z-Score
**1[a]**	**1.86**
**2[a]**	**0.95**
3	-0.36
4	-0.81
5	-0.44
6	-1.87
**7[a]**	**0.53**
8	0.25

[a] Green marked cavities represent the considered pockets.

Table S4. Results of Cavity Detection by MOLCAD.

Surface	Volume/Angstrom^3	Docking Score
1	419.329	5.5540
2	318.249	4.5826
3	247.439	3.4005
4	243.154	6.6469
5	211.727	4.3751

The order is according to the size of the volume.

Table S5. Behavior of the PG at Each Binding Site during MD Simulation

Binding site	Residence time (ps)	
	Lipid11	Lipid21
I	13147	18482
II	>25000	>25000
III	3793	2405

Table S6. The Calculated Time that PGs remained stable at site II

Length of C- chain	Stable time (ps)	BFE kcal/mol	Length of C- chain	Stable time (ps)	BFE kcal/mol
4	1960	-40.30	12	23863	-62.68
6	>50000	-47.82	14	31334	-62.11
8	31051	-27.14	16	33234	-53.53
10	>50000	-55.61	18	34575	-64.83

Table S7. The Distribution of The Key Interaction Residues with PG in Site II

Force Field	Key Residues
Lipid11	Ile229, Tyr306, Pro307, Tyr333, Thr335, Lys336, Gly337, Arg347, Tyr363, Lys368
Lipid21	Ile229, Tyr306, Pro307, Tyr333, Thr335, Lys336, Gly337, Ser365, Asn367, Lys368

Table S8. Binding Free Energies for the LPOR-PG Complexes Calculated from Independent MD Trajectory with Lipid11 and Lipid21 Force Fields [a]

System	Ele	VdW	Pol	Non-Pol	Total	Stable time(ps)
Lipid11						
LPOR/PG-4C	-162.5	-44.3	172.9	-6.4	-40.3	1960
LPOR/PG-10	-183.2	-59.0	194.2	-7.6	-55.61	50000
LPOR/PG-14	-192.4	-62.0	200.4	-8.1	-62.11	31334
LPOR/PG-18	-204.2	-77.9	225.8	-8.5	-64.83	34575
Lipid21						
LPOR/PG-4C	-156.6	-40.4	169.9	-5.6	-32.7	1874
LPOR/PG-10	-168.9	-61.4	187.8	-8.3	-50.9	50000
LPOR/PG-14	-183.6	-70.4	193.5	-10.0	-70.5	42152
LPOR/PG-18	-194.7	-69.2	191.9	-9.9	-81.9	46266

[a] All values are given in kcal/mol.

Table S9. Decomposition for the Important Residues Contributing to the Binding Free Energy [a]

System	Residue	Ele	VdW	Pol	Non-Pol	Total
LPOR/PG-10C-Lipid11	Lys368	-49.13	-3.50	48.86	-0.47	-4.24
	Lys336	-20.40	-4.87	23.41	-0.34	-2.20
	Tyr306	-11.80	-1.90	12.62	-0.30	-1.38
	Ile229	-7.54	-1.05	7.67	-0.26	-1.18

LPOR/PG-10C-Lipid21	Lys368	-47.21	-4.11	46.65	-1.01	-5.68
	Lys336	-23.5	-4.04	24.62	-0.35	-3.27
	Tyr306	-10.44	-1.3	10.37	-0.28	-1.65
	Ile229	-9.1	-1.1	9.38	-0.27	-1.09

[a] All values are given in kcal/mol.

Table S10. The MM/GBSA binding free energy calculation for PG with different states

State	Energy/(kcal/mol)				
	van der Waals	Electrostatic	Polar Solvation	Non-Polar Solv	Total
MES	-24.72	-73.97	83.25	-3.28	-18.72
TES	-59.85	-160.42	180.78	-7.82	-47.31

Table S11. Basic information of the final clusters of the LPOR/NADPH/PG structures obtained from the aMD simulation

#Cluster	Frames	Frac/%	Centroid	AvgCDist /Å	N@R346-P@PG AvgCDist/Å
1	1181	9.45	8566	5.00	20.56
2	1066	8.53	7894	4.11	20.32
3	1025	8.20	6874	3.44	21.48
4	894	7.15	6200	3.22	19.98
5	883	7.06	8954	2.77	22.03
6	825	6.60	6679	2.77	18.45
7	819	6.55	10445	3.63	19.56
8	815	6.52	5357	3.44	16.30
9	798	6.38	9855	4.11	19.62
10	776	6.21	5743	5	19.10
11	770	6.16	8655	4.49	20.03
12	775	6.2	10045	4.23	20.94
13	749	6.04	1673	4.91	17.24
14	350	2.8	4890	4.01	15.34
15	327	2.8	3500	4.32	15.56
16	125	1.0	4450	3.76	14.38
17	113	0.9	1840	4.57	10.5
18	100	0.8	2234	4.41	10.38
19	25	0.2	2679	4.16	10.07
20	22	0.2	1461	3.36	9.55
21	13	0.1	1616	3.21	10.24
22	13	0.1	3428	3.13	8.07
23	12	0.1	2324	3.28	8.44
24	5	0	174	3.36	10.25
25	4	0	187	3.45	11.11

Notes: (1) Due to the current limitation of the calculation, a frame is extracted every 2 ns here, and a total of 12500 frames were included in the cluster analysis. (2) When the distance N@R346-P@PG is greater than 15 Å, it indicates that the PG has left the site II. The corresponding entries extracted in Figure 12B are colored in red.

Table S12. Basic information of the final clusters of the LPOR/PG/NADPH structures obtained from aMD simulation

#Cluster	Frames	Frac/%	Centroid	AvgCDist /Å	N@R346-P@PG AvgCDist/Å
1	1128	9.17	9333	4.73	22.36
2	819	9.02	8486	4.15	21.98
3	811	6.55	9985	4.47	19.36
4	803	6.49	10337	4.86	20.65
5	759	6.42	8487	3.32	19.58
6	754	6.07	5846	3.36	15.99
7	750	6.03	7833	4.55	16.83
8	736	6	8719	3.54	21.41
9	596	5.89	9441	4.42	19.07
10	593	4.77	7666	4.02	18.02
11	581	4.74	8658	3.87	19.72
12	579	4.65	5666	4.03	17.17
13	524	4.63	8985	4.79	13.51
14	348	4.19	4970	3.22	12.45
15	334	2.78	5453	3.04	16.22
16	329	2.67	4098	5.00	9.54
17	263	2.63	3109	3.99	8.61
18	260	2.1	4688	4.48	11.52
19	253	2.08	2083	3.88	10.25
20	75	2.02	3391	4.20	14.36
21	63	0.6	1302	4.78	9.32
22	8	0.5	5174	4.38	18.48
23	4	0	5766	3.80	19.09
24	4	0	2333	4.99	10.36
25	2	0	8486	4.15	16.98

2. Supplementary Figures

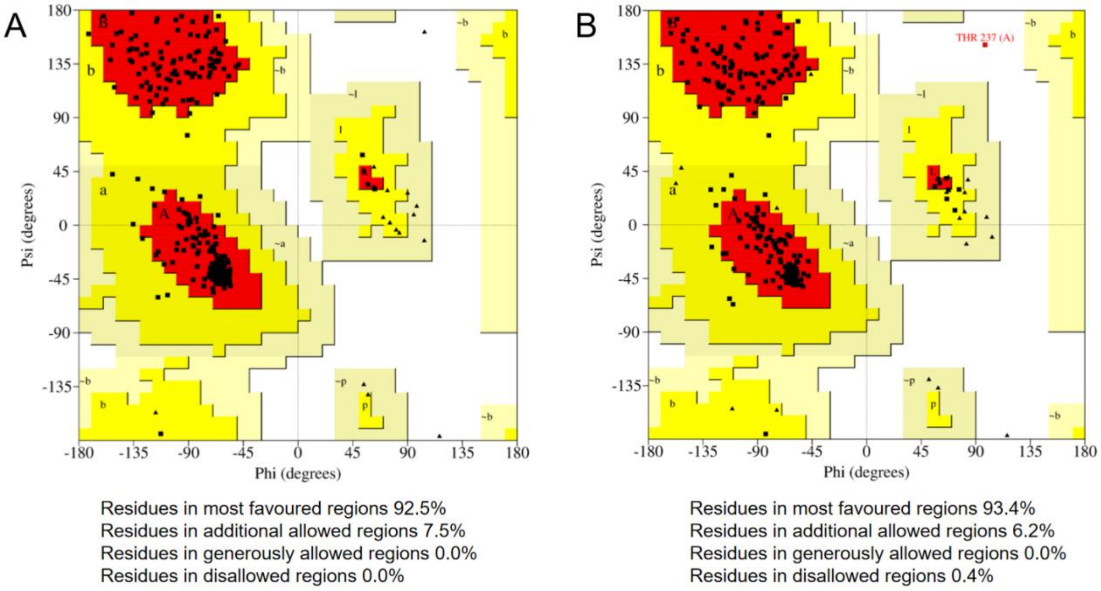

Figure S1. Ramachandran Plot of (A) template protein (PDBID:6l1h) and (B) predictor protein.

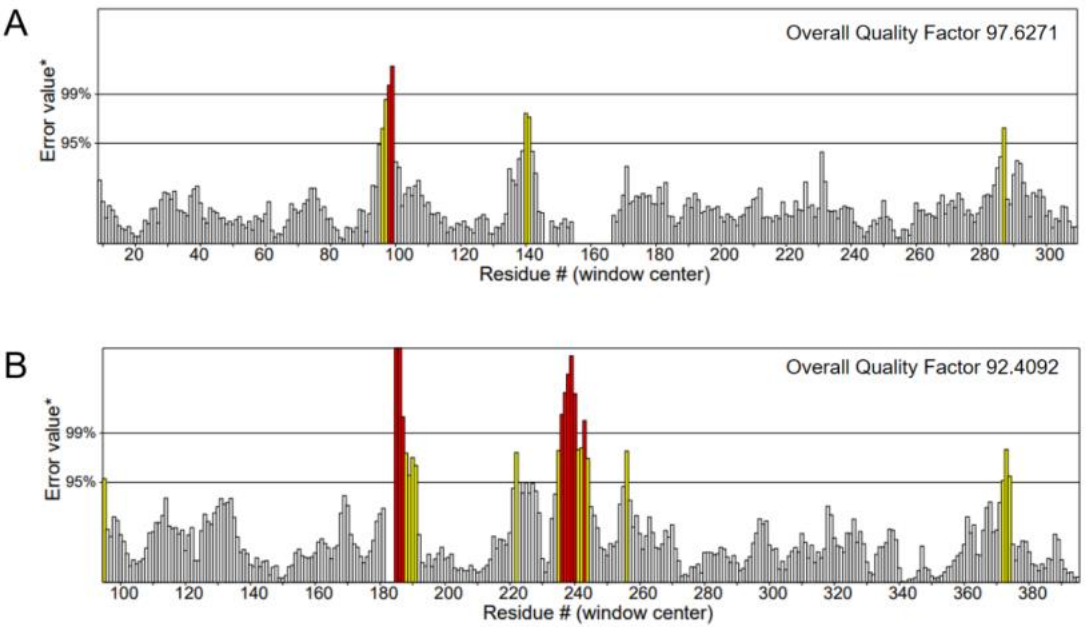

Figure S2. ERRAT of (A) template protein (PDBID:6l1h) and (B) predictor protein.

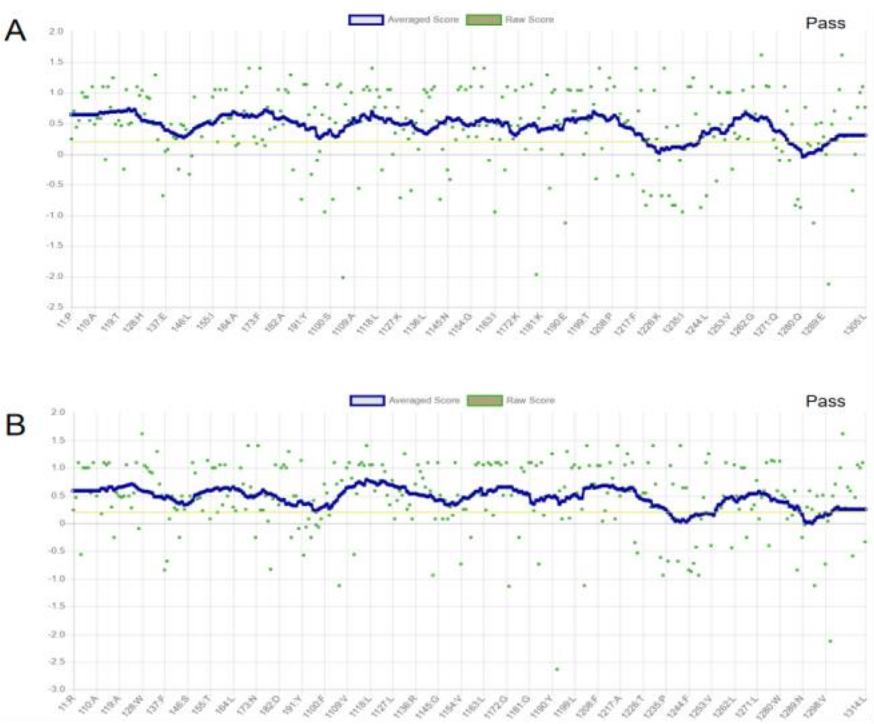

Figure S3. VERIFY3D of (A) template protein (PDBID:6l1h) and (B) predictor protein. Respectively, 88.20% and 90.45% of the residues have averaged 3D-1D score0.2.

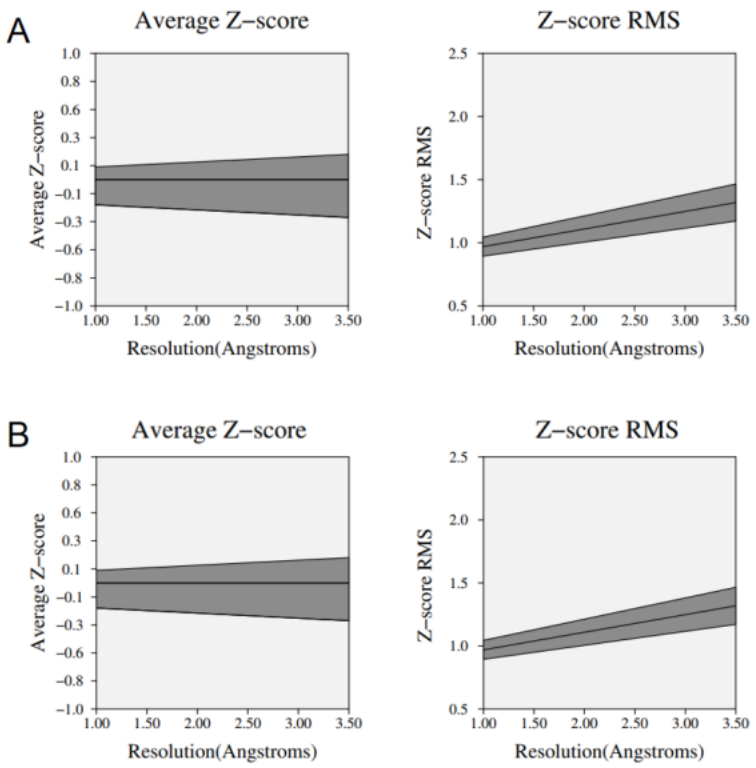

Figure S4. PROVE of (A) template protein (PDBID:6l1h) and (B) predictor protein. Respectively,98.7% and 95.9% protein atoms are considered as usual stereochemistry.

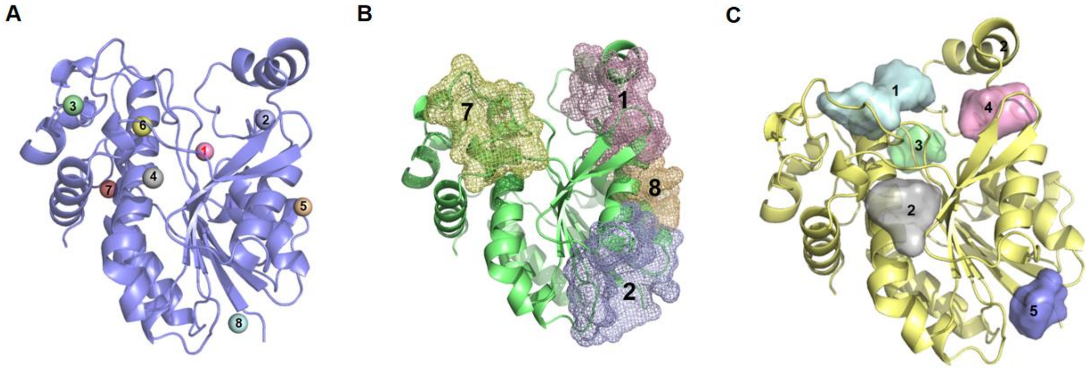

Figure S5. Results of Cavity Prediction by (A) PARS, (B) Corrsite, and (C) MOLCAD.

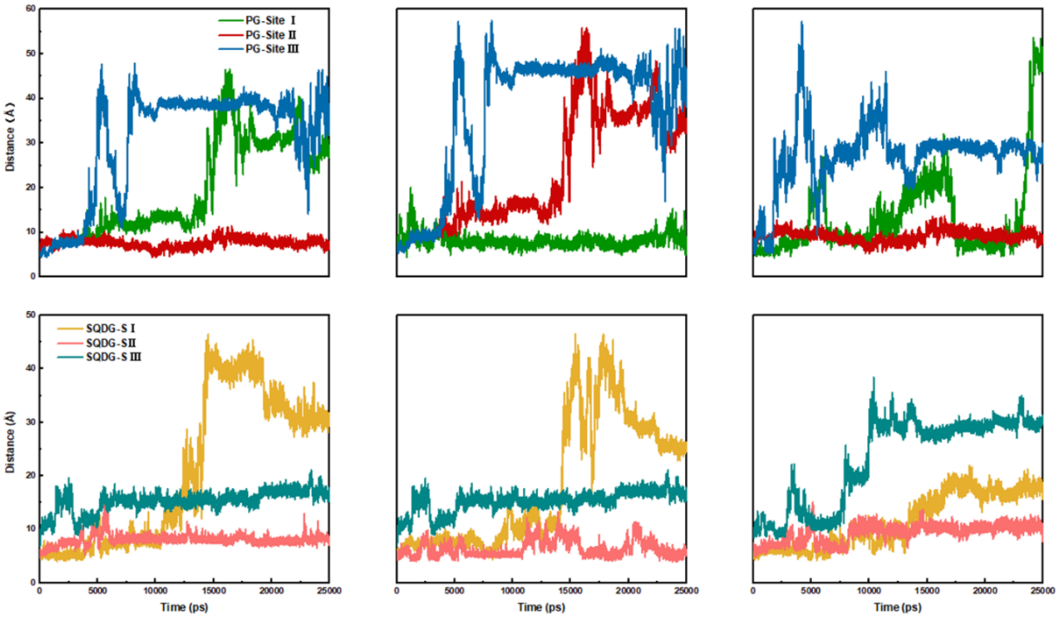

Figure S6. The distance between two LPOR regulators and each binding site during the MD Simulation time.

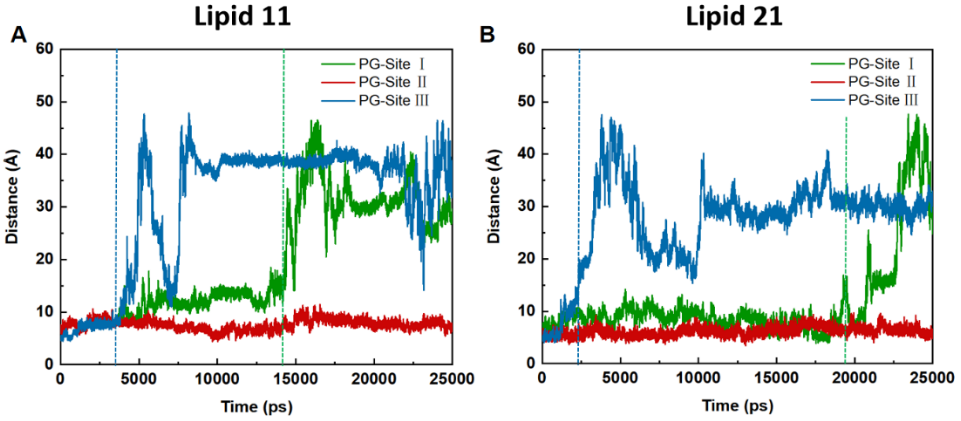

Figure S7. The distances between PG and site I, II and III
in molecular dynamics simulation with Lipid 11 (A) and Lipid 21(B) force fields.

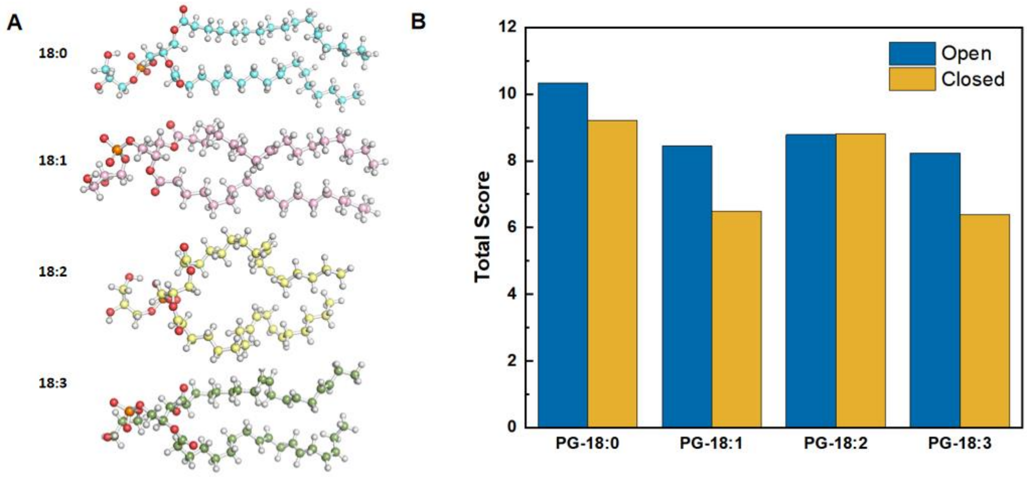

Figure S8. (A) The constructs of PGs with a different index of unsaturated fatty acid (IUFA) before docking. (B) Docking results for different PGs to open and closed LPOR.

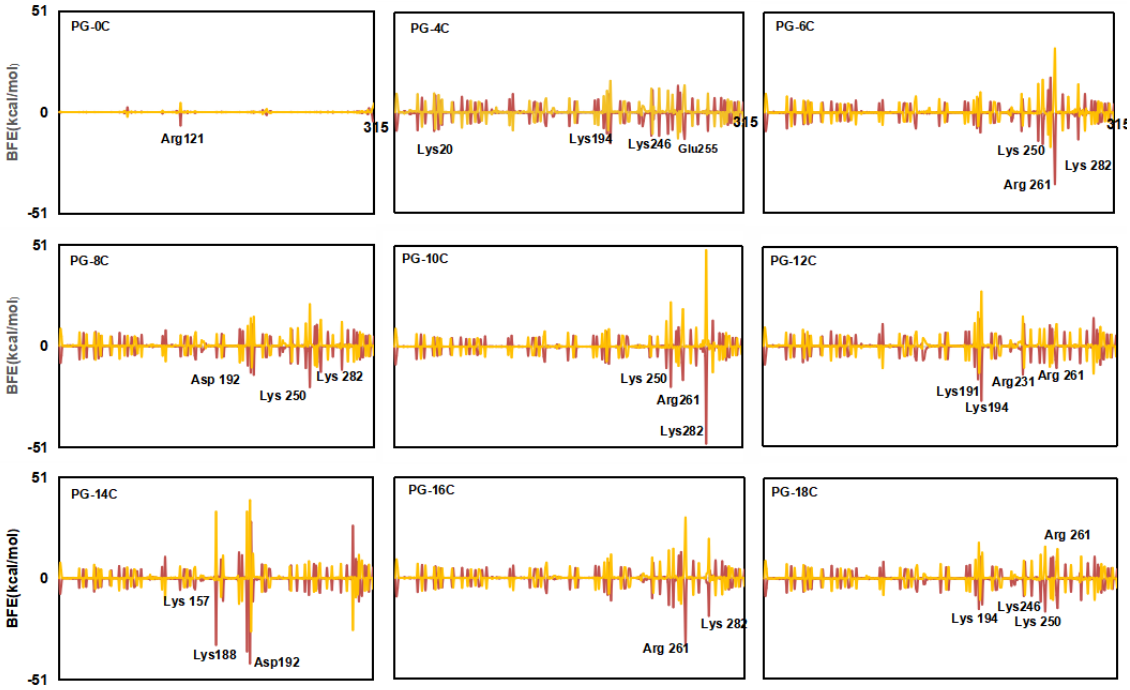

Figure S9. Energy decomposition in nine systems. The electrostatic and polar solvation energy are shown as red and yellow line, respectively. Residue numbering starts from the removal of the extra 86 residues at the C-terminus.

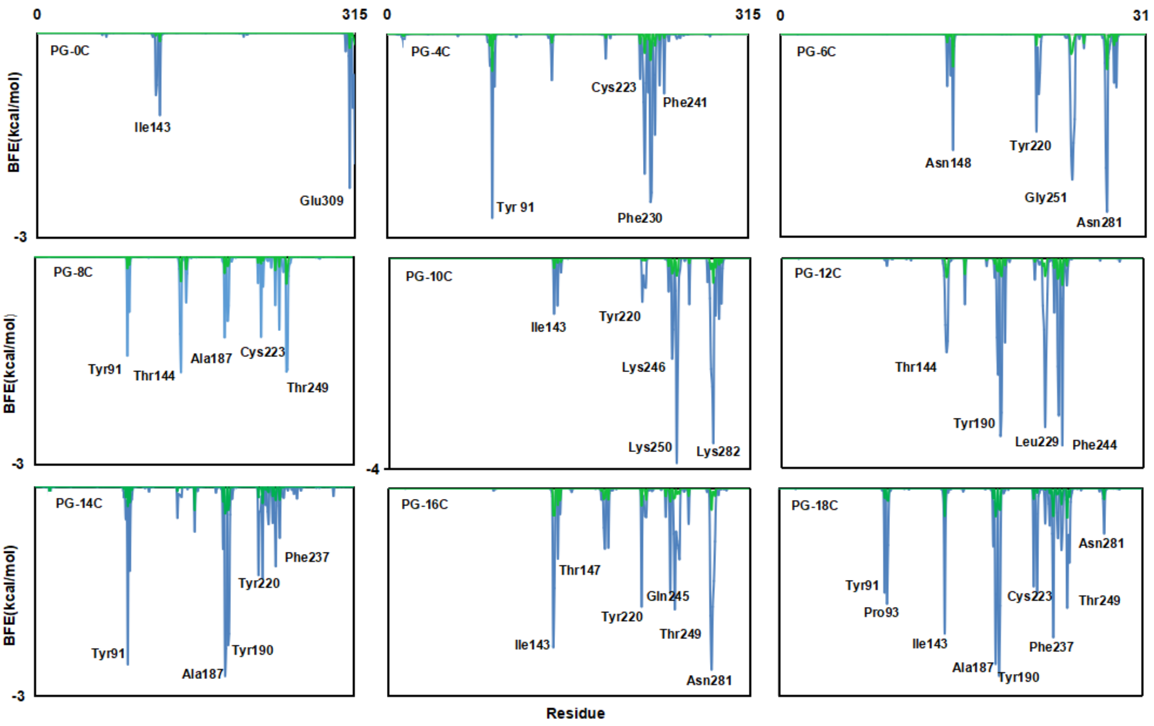

Figure S10. Energy decomposition in nine systems. The van der Waals and Non-polar solvation energies are shown as blue and green lines respectively. Residue numbering starts from the removal of the extra 86 residues at the C-terminus.

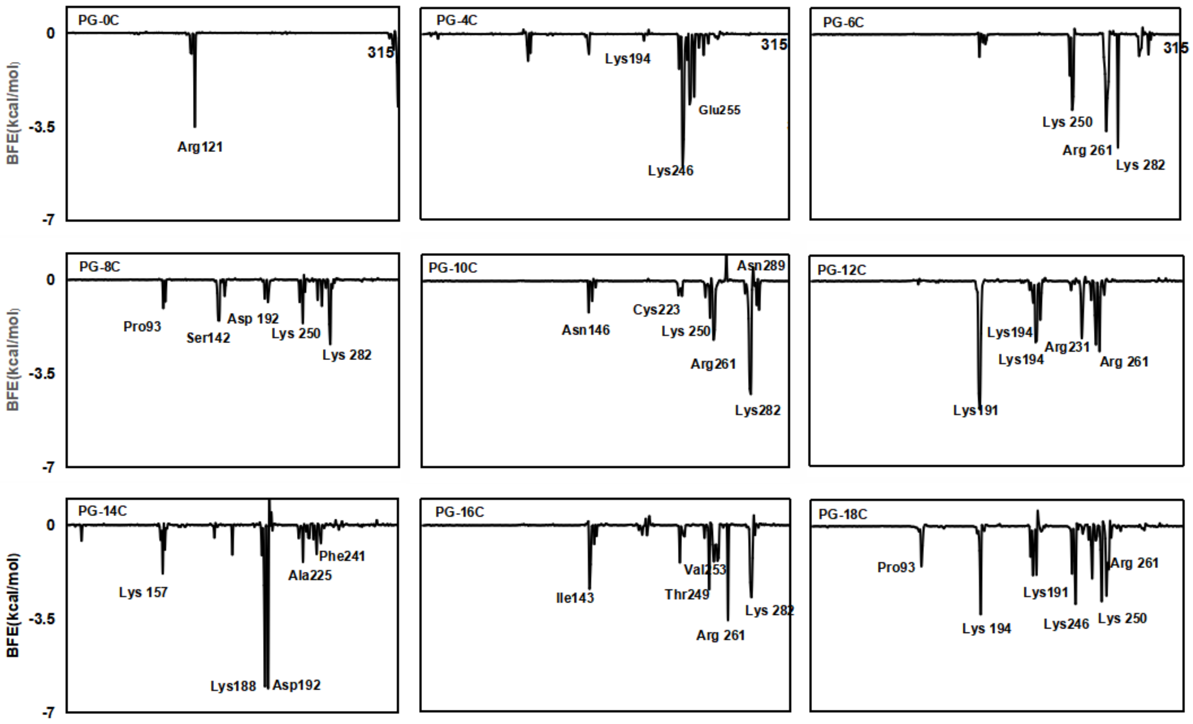

Figure S11. Decomposition of total energy in nine systems. Residue numbering starts from the removal of the extra 86 residues at the C-terminus.

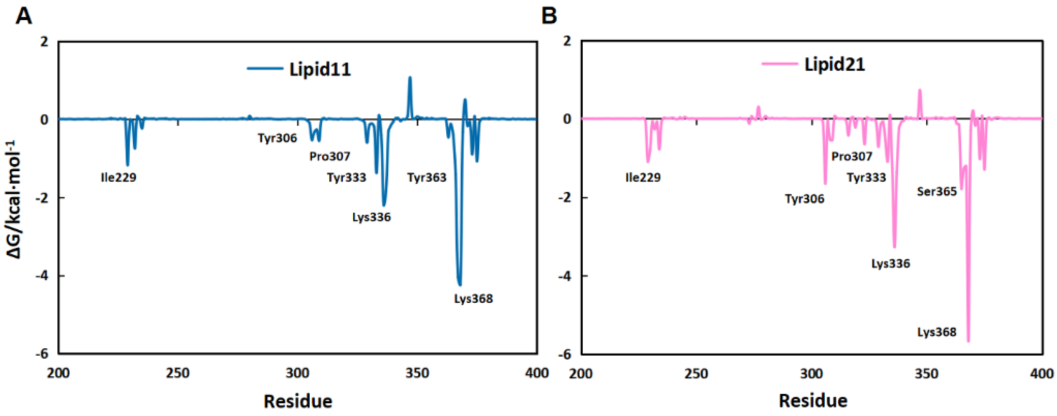

Figure S12. Total binding free energy contributions of LPOR-PG complexes. Each residue for the LPOR-PG complexes calculated from the equilibrated conformations during independent MD run with (A)Lipid11 and (B)Lipid21 force fields.

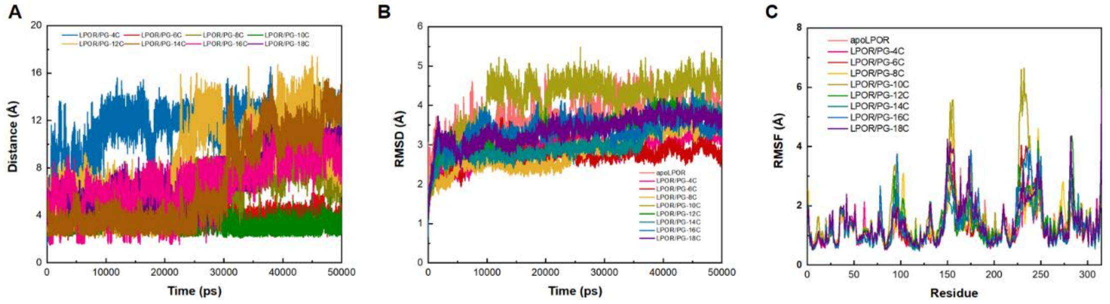

Figure S13. (A)The distance between PG and site II in aMD. Only PG-6C and PG-10C bind stably at the allosteric site, and LPOR/PG-8C system begins to dissociate from the site after 20 ns. All other PGs leave the site quickly. (B) The RMSD and (C) RMSF curves for LPOR/PGs systems. The LPOR/PG-10C exhibits the best activation characteristics.

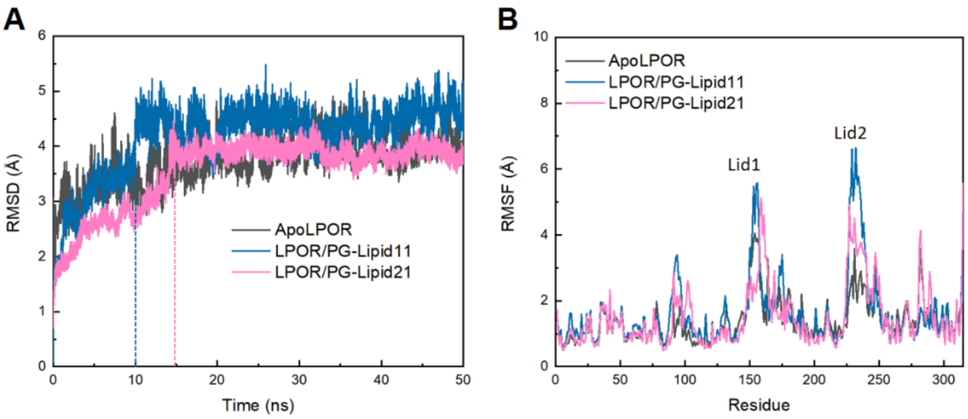

Figure S14. (A)The time-evolution RMSD curves of LPOR and LPOR-PG complex under Lipid11 and Lipid21 force fields. (B) The RMSF curves of LPOR and LPOR-PG complex calculated from independent accelerated MD simulation with two different force fields.

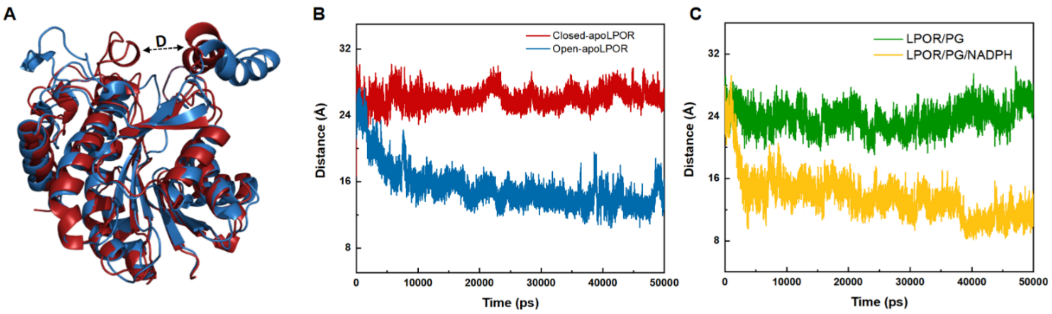

Figure S15. (A) Superimposed views of open and closed LPOR. The distance between Leu 236 and Thr335 to characterize the opening degree of the lid. (B) The change in the distance in 50ns simulation. (C) The change in the distance in binary (LPOR/PG) and ternary (LPOR/PG/NADPH) complexes. The above simulations all start from the open LPOR to improve the efficiency of obtaining stable binding of PG to the protein.

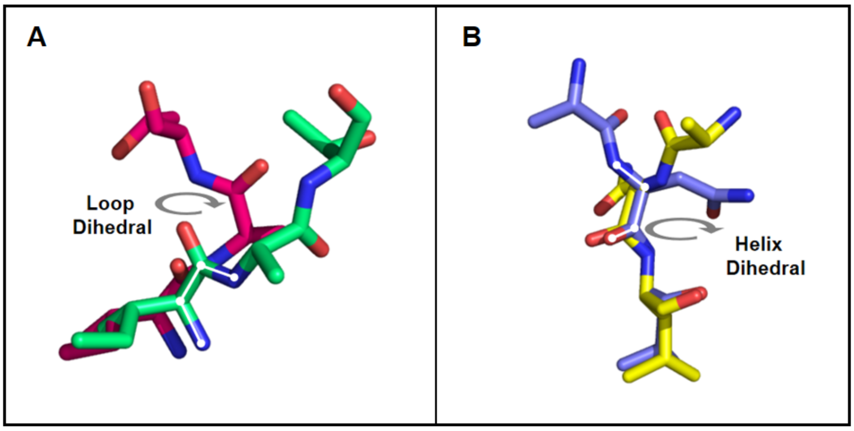

Figure S16. (A)Conformational flipping of the I229-T230-G231 and 310I-311A-312T rotary microswitches at the initial and final states in the simulations. The two dihedral angles correspond to dihedral angles 1 and 2, respectively.

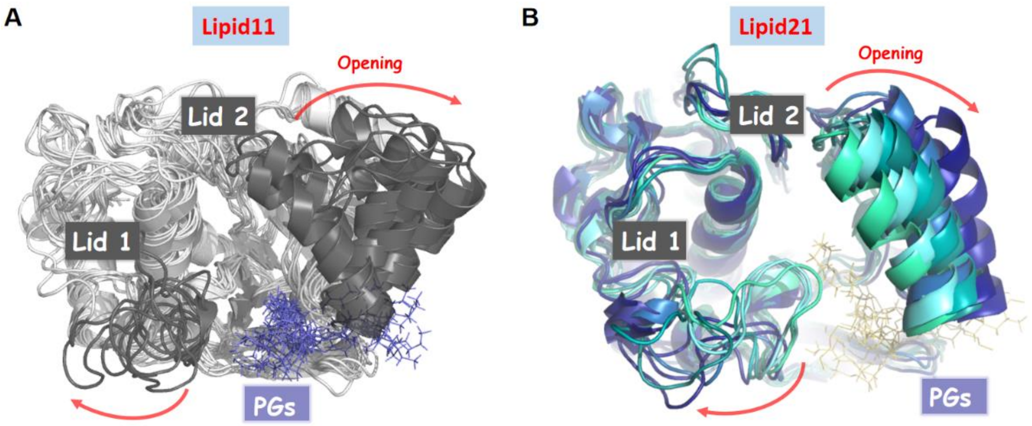

Figure S17. The integral opening process shown to emphasize the hinge and lid motion in the simulated close LPOR structure under two different force fields, viewed from above.

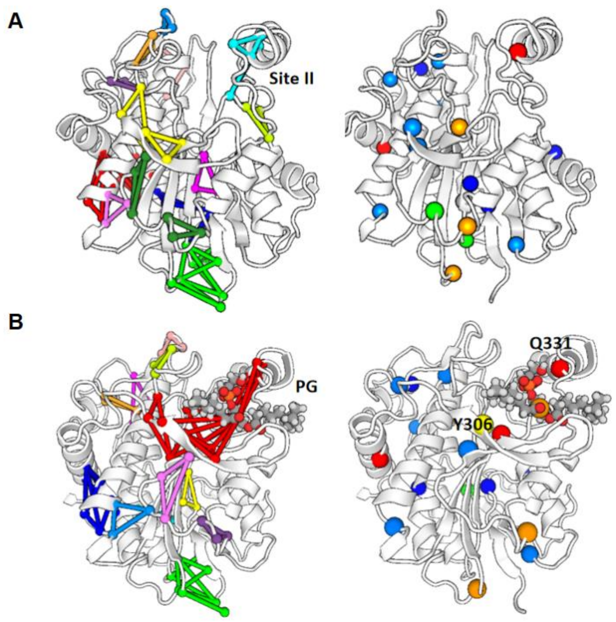

Figure S18. The community(left) and hub(right) structures of residue contact networks in (A) unbound LPOR and (B)LPOR bound with PG. The community and hub structure describe the fundamental feature of residue contact networks.

Community	Nodes	Links	Hubs	Community	Nodes	Links	Hubs
1	10	14	3	1	16	26	5
2	9	12	1	2	9	12	1
3	6	7	3	3	7	10	2
4	5	7	2	4	3	3	0
5	3	6	1	5	3	3	0
6	3	3	1	6	3	3	0
7	3	3	0	7	3	3	0
8	3	3	0	8	3	3	0
9	3	3	0	9	3	3	1
10	3	3	0	10	3	3	0
11	3	3	1	11	3	3	1
12	3	3	0	12	3	3	0
13	3	3	2				
14	3	3	0				

apoLPOR
LPOR/PG

Figure S19. The community of residue contact networks in unbound LPOR and LPOR bound with PG.

Index	Hub	Avg Force	Num of Links	Community	Index	Hub	Avg Force	Num of Links	Community
1	V92	4.0075	4	2	1	T94	4.5825	4	2
2	T94	4.5825	4	2	2	W114	5.8880	5	2
3	W114	5.8880	5	2	3	K125	4.9875	4	2
4	K125	4.9875	4	2	4	L145	6.2125	4	2
5	L145	6.2125	4	2	5	F191	5.1340	5	2
6	L167	6.2950	4	0	6	L204	4.2300	4	0
7	L170	3.2060	5	2	7	Y218	8.0650	6	2
8	V171	4.190	4	2	8	L246	5.3725	4	2
9	F191	5.1340	5	0	9	M264	4.2075	4	0
10	L204	3.9800	5	2	10	K274	5.9200	4	2
					11	F294	5.6600	4	2
					12	Y306	10.2340	5	0
					13	Q331	9.3780	5	2
					14	D354	8.5200	4	0
					15	Y363	5.9940	5	0
					16	N375	4.4560	5	4
					17	W391	10.7043	7	4
					18	A402	8.0075	12	0

apoLPOR
LPOR/PG

Figure S20. The hub of residue contact networks in unbound LPOR and LPOR bound with PG.

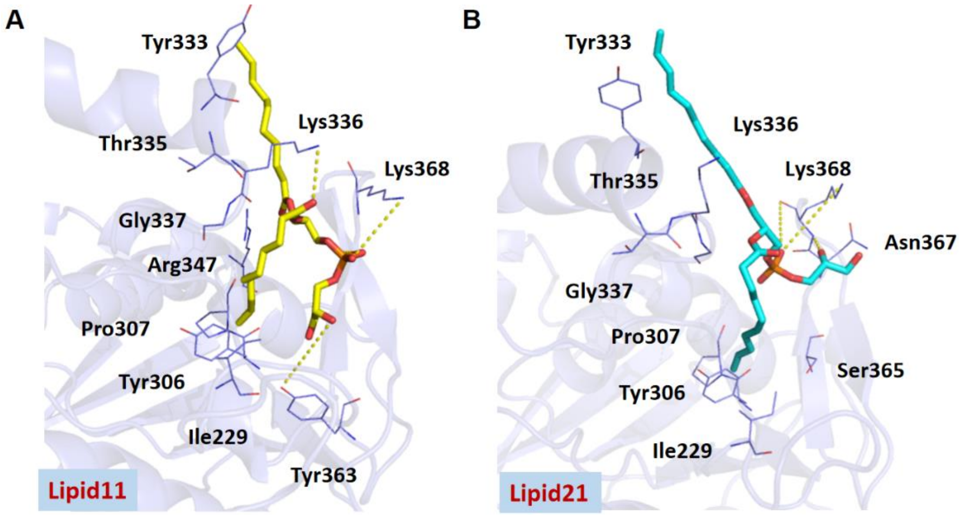

Figure S21. Key interactions at the active sites of the representative conformations of LPOR-PG complexes with equilibrium stabilization in (A)Lipid11 and (B)lipid21 force fields respectively.

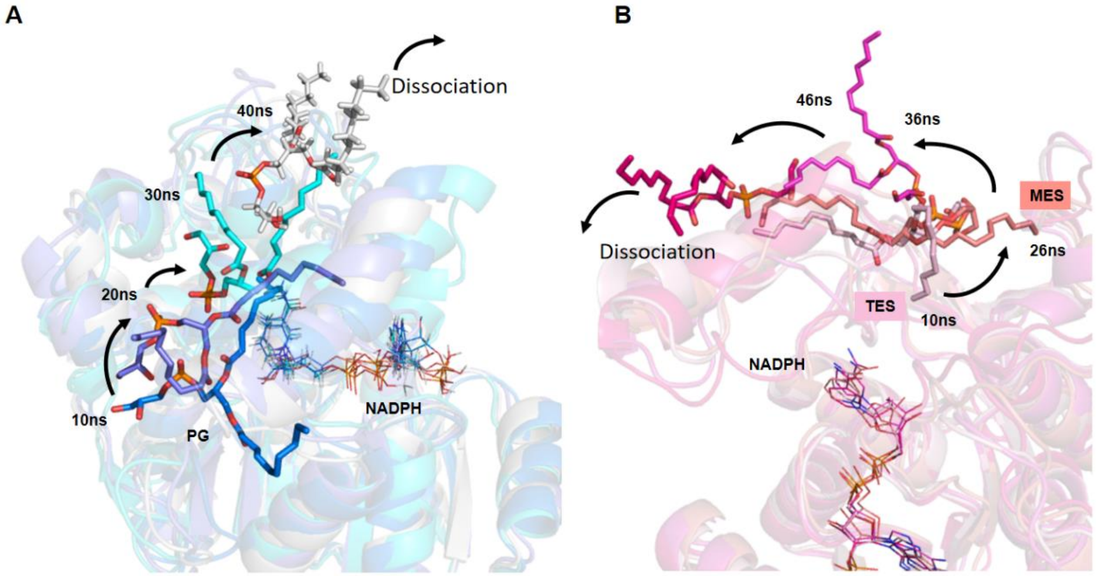

Figure S22. The extracted conformation in the two simulations. The NADPH (A) or PG (B) preferentially combined with LPOR respectively
